# A hot origin of dissimilatory sulfite reduction catalyzed by DsrAB in the Paleoarchean Era

**DOI:** 10.1002/mlf2.70066

**Published:** 2026-02-23

**Authors:** Lingyun Tang, Zhenhao Luo, Shaoming Gao, Zhiliang Lin, Mengqi Sun, Runsheng Li, Shu‐Hong Gao, Geng Wu, Yiliang Li, Linan Huang, Lu Fan

**Affiliations:** ^1^ Department of Ocean Science and Engineering Southern University of Science and Technology (SUSTech) Shenzhen China; ^2^ State Key Laboratory of Biocontrol, Guangdong Provincial Key Laboratory of Plant Resources and Southern Marine Science and Engineering Guangdong Laboratory (Zhuhai), School of Life Sciences Sun Yat‐Sen University Guangzhou China; ^3^ School of Life Sciences Sun Yat‐Sen University Guangzhou China; ^4^ Department of Infectious Diseases and Public Health, Jockey Club College of Veterinary Medicine and Life Sciences City University of Hong Kong Hong Kong China; ^5^ State Key Laboratory of Urban Water Resource and Environment, School of Civil & Environmental Engineering Harbin Institute of Technology Shenzhen China; ^6^ State Key Laboratory of Geomicrobiology and Environmental Changes China University of Geosciences Wuhan China; ^7^ Department of Earth Sciences The University of Hong Kong Hong Kong China

**Keywords:** archaeal evolution, *dsrAB*, metagenomics, sulfite reduction, sulfur cycling

## Abstract

Dissimilatory sulfite reduction (DSR) has been essential to microbial energy metabolism in the biogeochemical sulfur cycle since the Paleoarchean Era. However, due to the lack of an integrated assessment of geological record and genomic data, the evolutionary origin of DSR remains elusive in terms of time, habitat, and genetic basis. In this study, we reconstructed the evolutionary pathways and the ancestral sequences of Dsr proteins by mining metagenomes ranging from mesothermal to hyperthermal environments. A phylogenetic analysis of the key catalytic enzyme, DsrAB, and other Dsr proteins indicates that the earliest and most basic functional cascade, DsrABCNM, emerged prior to the latest common ancestor of several basal branching DsrAB clusters encoded by bacteria and archaea. Using a molecular dating strategy that calibrates the protein tree with a species tree, we predicted that the DSR originated 3.508 billion years ago (Ga). This finding strongly confirms the earliest geological evidence of DSR ( ~ 3.47 Ga). Further predictions from ancestral sequence reconstruction indicate that the optimal catalytic temperature of DsrA at the time of DSR origin was approximately 73°C, which is consistent with the petrographic and geochemical evidence in early Archean hydrothermal deposits. After its hot origin, DsrA diversified into subclades that adapted to various temperature levels following the Great Oxidation Event. This is exemplified by the evolution of the reductive archaeal‐type DsrA. Our results synchronize the molecular ages with the geological record, which advances our understanding of the earliest DSR systems and highlights the enzymatic adaptations of microbial life in the Archean biosphere.

## Impact statement

Sulfite reduction was an essential biogeochemical process on early Earth, but its evolutionary origin remains unclear. By developing a new molecular dating strategy, we estimate that the ubiquitous dissimilatory sulfite reductase DsrAB originated more than 3.508 billion years ago (Ga). This finding provides strong support for the oldest geological record of dissimilatory sulfite reduction (~3.47 Ga), which was discovered over two decades ago. Furthermore, ancestral protein reconstruction suggests that the first sulfite‐reducing microorganisms were likely thermophiles or moderate thermophiles, a conclusion consistent with geological evidence from Archean hydrothermal deposits.

## INTRODUCTION

Sulfur played a vital role in the evolution of early life. H_2_S and SO_2_ were released into the Earth's surface by volcanic activity, providing the primary source of sulfur‐containing compounds in microbial metabolism on early Earth^1^, ^2^. Modeling studies have also suggested that sulfites may have been presented at concentrations equal to or greater than those of sulfate in oxidant‐poor water prior to the Great Oxidation Event (GOE)^3^, ^4^. In such an anoxic environment, microorganisms could reduce these widely available compounds, such as (thio)sulfate and sulfite, for anaerobic respiration^5^, contributing to the earliest biogeochemical sulfur cycle on our planet^6^. However, the origin of dissimilatory sulfite reduction (DSR) in microorganisms is not fully understood, including its geological time, environmental conditions, and the catalytic proteins involved.

Sulfur isotopic data suggest that sulfite reduction should have appeared before 3.5 Ga^2^, ^7^. The discovery of sulfide in the barite deposits of the “Dresser Formation” in Australia is the earliest evidence of DSR; however, the ambient temperature of this bioactivity is debated^8^. Using the methods of quadruple isotope systematics and interfacial angles, researchers concluded that these sulfate/sulfite reduction microorganisms were likely mesophiles (i.e., organisms that thrive in mesothermal environments—ambient temperature from 15°C to 45°C) or moderate thermophiles (i.e., organisms that thrive in moderate thermal environments—ambient temperature from 45°C to 60°C)^8^, ^9^. However, a recent study of stromatolites, microbial palisade fabric, and gas bubbles revealed that the Dresser Formation layer was originally from terrestrial hot spring areas^10^. Therefore, it remains unclear whether the earliest DSR occurred only in the mesothermal (15°C to 45°C) to moderate thermal (45°C to 60°C) outskirt environments of the springs or also at the hot (60°C to 80°C) to hyperthermal (>80°C) spring vents.

On the other hand, genomic and phylogenetic analyses have yet to conclude on the ambient temperature during the earliest stage of DSR. The enzymes currently known to be involved in DSR include dissimilatory sulfite reductase (Dsr), anaerobic sulfite reductase, and cytochrome *c* sulfite reductase^11^, ^12^, ^13^, ^14^, ^15^, ^16^, ^17^. Whereas the latter two are sporadically encoded by bacteria^15^, Dsr enzymes are widely distributed among bacteria and archaea. They are thought to be among the earliest enzymes to play an essential role in microbial energy metabolism on early Earth^8^, ^16^. Previous phylogenetic analyses have generally assigned homologs of the two essential proteins in the Dsr cascade, Dsr subunits A and B (DsrAB), to four major subclades: the basal‐type DsrAB (function unknown), the reductive archaeal‐type DsrAB (RA‐DsrAB, which catalyzes sulfite to sulfur reduction and is exclusively found in Archaea), the reductive bacterial‐type DsrAB (which catalyzes sulfite to sulfur reduction and is exclusively found in Bacteria, with very few exceptions), and the oxidative bacterial‐type DsrAB (which catalyzes sulfur to sulfite oxidation and is exclusively found in Bacteria)^11^, ^12^, ^15^, ^16^, ^18^. Since there is still a lack of empirical validation that basal‐type DsrAB homologs function as sulfite reductases, RA‐DsrAB has been identified as the earliest form of functional Dsr^16^, ^17^, ^19^. Whereas RA‐DsrAB have been previously mainly found in *Thermoplasmatota* (formerly known as *Euryarchaeota*) and *Thermoproteota* archaea from hydrothermal environments^17^, ^18^, ^20^, ^21^, ^22^, ^23^, ^24^, recent metagenomic approaches have detected archaeal lineages such as *Nitrososphaeria* (formerly known as *Thaumarchaeota*) and *Thermoplasmatota*, which carry *dsrAB* genes in mesothermal environments, including acid mine drainage (AMD)^25^, ^26^. This leaves the ambient temperature at the origin of the DSR still an open question.

Furthermore, the modulation trajectory of proteins in the early evolutionary history of the Dsr cascade remains unclear. DsrAB is a heterotetrameric complex that produces a DsrC‐trisulfide from sulfite and DsrC^27^. DsrC‐trisulfide is an intermediate that acts as the final acceptor of electrons from the DsrAB complex^12^. DsrN is necessary for aiding the siroheme cofactor in DsrAB, and it is highly conserved^28^. DsrM and DsrK form an electron‐donating membrane complex DsrMK, which catalyzes the reduction of DsrC‐trisulfide and releases sulfide and DsrC^29^. The protein combination DsrABCNMK is considered the minimal functional set of the Dsr cascade^28^. In a recent genomic and phylogenetic study^28^, this minimal set was first assembled in sulfite‐reductive archaea, and then transferred to sulfite‐reducing and sulfur‐oxidizing bacteria. Subsequently, with the acquisition of additional Dsr proteins, including the DsrJOP complex that binds to DsrMK, a membrane complex formed^28^, ^30^. DsrD regulates the DsrAB complex^31^, ^32^, DsrT regulates the expression of DsrMKJOP^15^, ^33^, and the DsrEFH complex acts as a sulfur donor for DsrC in sulfur‐oxidizing microorganisms^34^, ^35^. However, the primitive forms of the functional Dsr cascade that predate the archaeal‐type DsrAB are still unknown.

To investigate the origin and early evolution of DSR, we conducted a large‐scale evolutionary analysis of DsrAB homologs, focusing on basal and archaeal DsrAB types close to the root of the DsrAB phylogenetic tree. We analyzed the evolutionary route of DsrAB and other proteins in the Dsr cascade and conducted molecular dating to estimate the time of DSR origin in the DsrA tree. Additionally, we reconstructed ancestral sequences of DsrA to predict the optimal catalytic temperature (*T*_opt_) of the enzyme throughout the evolutionary tree. We then contrasted the findings of the molecular evolutionary analysis with geological records to verify the consistency of the timing and environments of DSR origin.

## RESULTS

### The evolutionary diversity of DsrAB

**Figure 1:**
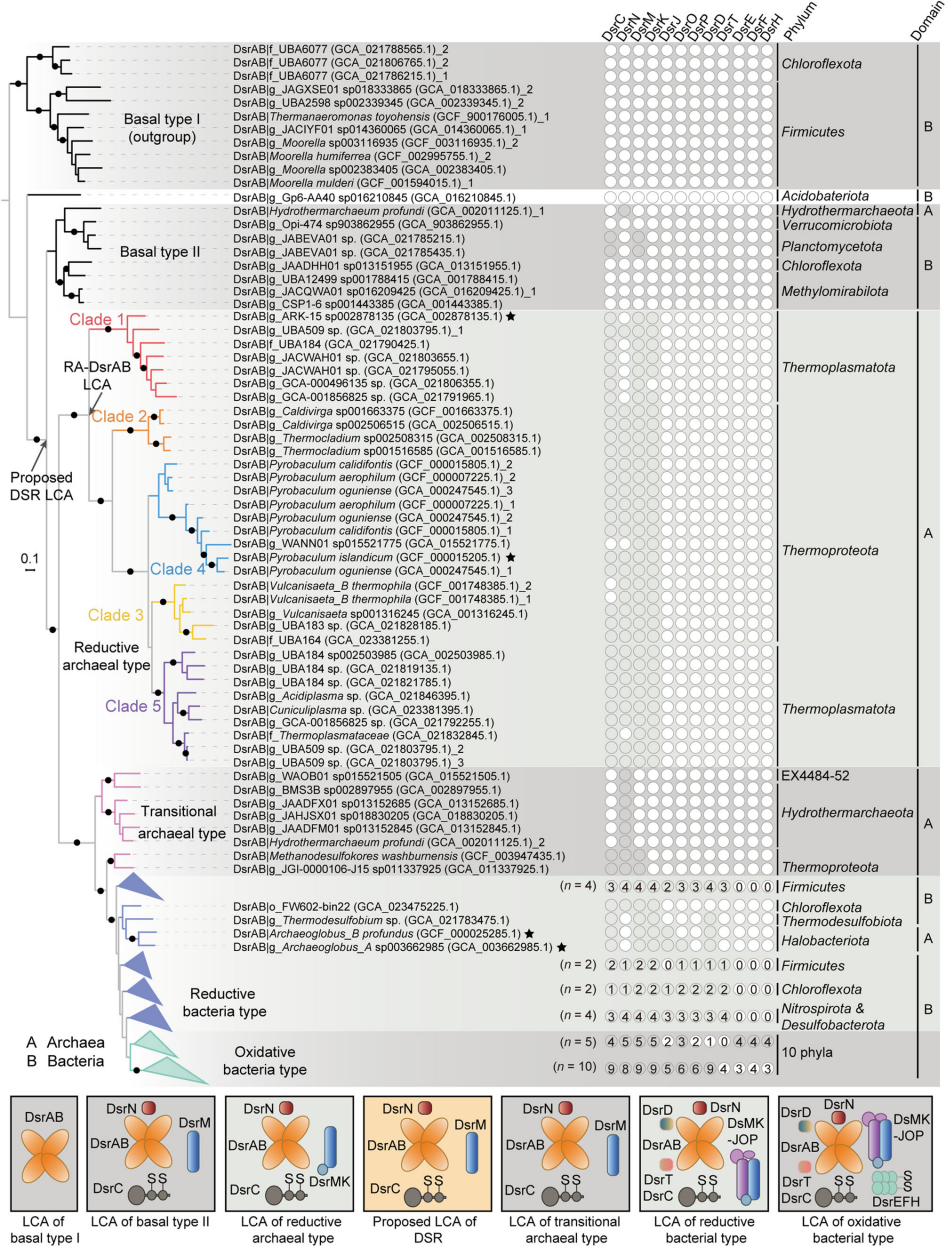
Phylogenetic analysis of DsrAB proteins and the origin of Dsr protein assemblies. The maximum likelihood phylogenetic tree (model automatically selected as LG + I + R9) of DsrAB (415 sequences, 970 alignment positions) is rooted by setting the clade of three *Chloroflexota* and eight *Firmicutes* DsrAB sequences (basal type I DsrAB) as the outgroup, according to the result of paralogous rooting. Branches with ultrafast bootstrap support values ≥95% are indicated by black dots. The branches of different types of DsrAB and the five monophyletic subgroups of RA‐DsrAB are colored differently. The presence of Dsr proteins in the genomes that encode DsrAB, and the Dsr proteins are placed in the same types of clusters in their specific phylogenetic trees, corresponding to the DsrAB types in the DsrAB tree (Figures S2–S11), shown as filled circles. Filled circles of folded branches of bacterial types indicate ≥50% cases of filled circles. The taxonomic classification is derived from the GTDB taxonomic ranking: A = Archaea, B = Bacteria, and “10 phyla” = *Chloroflexota*, *Proteobacteria*, *Spirochaetota*, *Actinobacteriota*, *Desulfobacterota*, *Nitrospirota*, SZUA‐79, SAR324, *Myxococcota*, and *Acidobacteriota*. The classification is derived from the GTDB taxonomic ranking, where “f_” and “g_” represent the classification of family and genus, respectively, and are used before the name of a candidate ranking. The asterisks indicate that the DSR functions of these DsrAB proteins have been experimentally verified^17^, ^39^, ^40^. The proposed combination of Dsr proteins for the latest common ancestor (LCA) of each DsrAB type is shown at the bottom of the figure.

A total of 415 unique DsrA‐DsrB concatenated sequences (hereafter referred to as DsrAB) were identified in 376 genomes, including 314 from the Genome Taxonomy Database (GTDB) v207_2^36^ and 62 from 92 AMD metagenomes (Table S1)^37^, ^38^. Phylogenetic inference using paralogous rooting revealed that the DsrAB homologs formed four major clusters. These clusters were named basal type I DsrAB, basal type II DsrAB, RA‐DsrAB, transitional archaeal‐type DsrAB, reductive bacterial‐type DsrAB, and oxidative bacterial‐type DsrAB (Figure 1). Basal type I contained DsrAB homologs of bacteria belonging to phyla *Chloroflexota* and *Firmicutes*. Basal type II DsrAB contained homologs of bacteria belonging to phyla *Verrucomicrobiota*, *Planctomycetoa*, *Chloroflexota*, and *Methylomirabilota*, and an archaeon of *Hydrothermoarchaeota profundi*. A basal branching DsrAB of *Acidobateriota* g_Gp6‐AA40 sp016210845 was found between these two DsrAB types. All homologs of RA‐DsrAB were from archaeal phyla *Thermoplasmatota* or *Thermoproteota*, and copies of the transitional archaeal type were from archaeal phyla EX4484‐52, *Hydrothermarchaeota*, and *Thermoproteota*. All DsrAB homologs of reductive bacterial type and oxidative bacterial type were from bacteria, except some homologs of the reductive bacterial type, which were from *Archaeoglobaceae*.

Whereas this topology of the DsrAB tree is consistent with previous studies^15^, ^17^, ^18^, ^28^, the updated GTDB database and the AMD data have provided us more details, particularly with regard to the basal and archaeal DsrAB types. For instance, three homologs of *Chloroflex* branching as sisters of *Firmicutes* and two bacterial DsrAB belonging to *Planctomycetota* were added as basal type II DsrAB. Moreover, the RA‐DsrAB diverged as a sister clade of basal type II DsrAB. Based on the tree topology and the results of pairwise sequence alignments, we divided the RA‐DsrAB sequences into five monophyletic clades by setting 60% as the protein identity threshold between clades (Figures 1 and S1). Sequences of clades 1 and 5 were all encoded by *Thermoplasmatota*, while all sequences in clades 2, 3, and 4 were from *Thermoproteota*. Clade 3 was restricted to the family *Thermocladiaceae* and clade 4 was restricted to the family *Thermoproteaceae*, suggesting taxonomy‐specific diversification of RA‐DsrAB (Table S1). Furthermore, the environmental abundances of these clades were correlated with various physicochemical parameters with statistical support (Supplementary Information).

### The evolution of the Dsr cascade assembly and the functional origin of DSR

To elucidate the evolutionary trajectory of the Dsr cascade assembly alongside the evolution of DsrAB, we reconstructed phylogenetic trees of Dsr proteins (Figure S2–S11 and Table S2–S3) and compared their topologies with those of DsrAB. If the topology of a Dsr protein tree follows the same pattern of the DsrAB tree as shown in Figure 1 (i.e., basal‐type DsrAB is the most deeply branched, followed by RA‐DsrAB, transitional archaeal‐type DsrAB, reductive bacterial‐type DsrAB, and oxidative bacterial‐type DsrAB), then the protein is considered to have an evolutionary link with DsrAB since its early diversification of DsrAB. Otherwise, the protein may have been recruited to the Dsr cascade during the later divergence of DsrAB (i.e., in the reductive bacterial and oxidative bacterial DsrAB types).

In trees of DsrC, DsrN, and DsrM, the homologs in the identical genomes of basal type II DsrAB, RA‐DsrAB, and transitional archaeal‐type DsrAB were clustered in monophyletic basal branching clades, with clear separation between the basal type II DsrAB and RA‐DsrAB (Figures S2–S4). This result suggests that in the latest common ancestor (LCA) of basal type II DsrAB, a minimum set of DsrABCNM might already exist (Figure 1). In the DsrK tree, homologs of RA‐DsrAB branched in a deep basal clade, suggesting that DsrK was likely recruited to the Dsr cascade in the LCA of RA‐DsrAB (Figures 1 and S5). After that, DsrD, DsrJ, DsrO, DsrP, and DsrT probably joined in the LCA of RA‐DsrAB, and DsrEFH was incorporated into the cascade at the LCA of the oxidative bacterial‐type DsrAB (Figures 1 and S6–S11).

Based on the likely patterns of the cascades in the LCAs of basal and archaeal DsrAB types, we infer a Dsr cascade pattern in the LCA of basal type II DsrAB and RA‐DsrAB homologs, with a minimum set of DsrABCNM (Figure 1). Despite the unresolved status of DsrK presence in this ancestral set, the comprehensive inclusion of all other key components of a functional DSR pathway is noteworthy. Furthermore, this configuration is distinctly different from that of the LCA of basal type I DsrAB lineages. Given the current absence of experimental evidence supporting the sulfite‐reducing activity of basal type I DsrAB copies^19^, ^41^, it is plausible that microbial DSR originated after the branching out of basal type I DsrAB but prior to the LCA of basal type II DsrAB and RA‐DsrAB (the node indicated in Figure 1). We refer to this LCA node as the LCA of DSR hereafter.

Regarding the placement of the DSR LCA in the DsrAB tree, the deepest‐branching clade predominantly comprises homologs that are currently encoded by bacteria (Figure 1). However, we cannot confirm whether the microbial host that encoded this DsrAB was a bacterium or an archaeon.

### The transfer of RA‐DsrAB‐encoding genes in archaea

**Figure 2:**
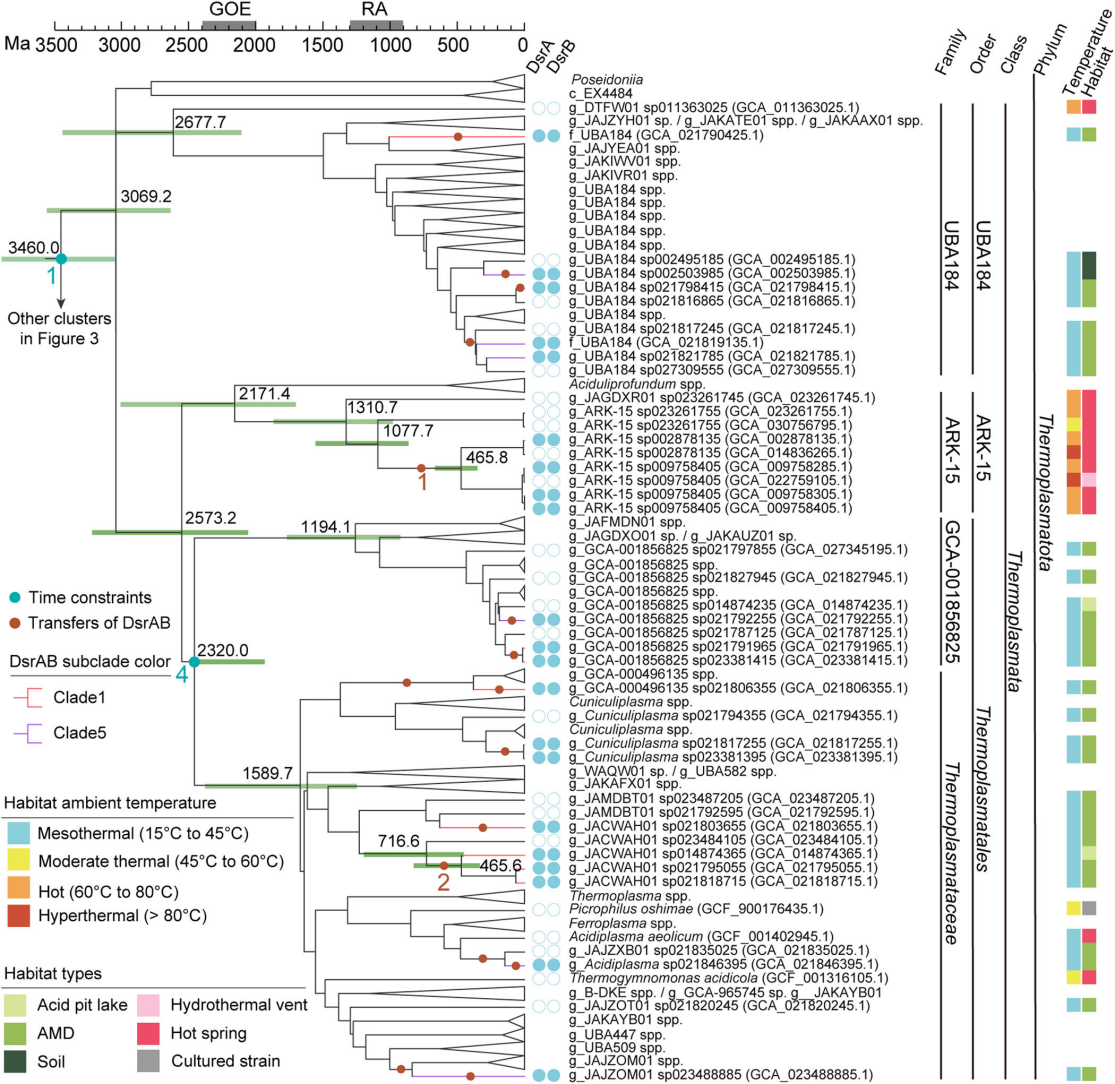
Evolutionary time scales and habitat feature of *Thermoplasmatota* encoding RA‐DsrAB. The maximum likelihood phylogenetic tree of archaea was reconstructed using 47 marker genes (model set up as LG + PMSF + F + G, with 12,166 alignment positions). The root was determined by setting the four bacterial genomes as the outgroup. Only the *Thermoplasmatota* clade of the tree is shown here, and the *Thermoproteota* clade is shown in Figure 3 for typeset need. Molecular dating was conducted using RelTime‐ML with the LG + G model, setting four node constraints, shown as numbers and blue dots on the respective nodes (Table S4). The geological time scale is shown above the tree. The period of the Rodinia Assembly (RA) and the Great Oxidation Event (GOE) are illustrated in the geological time scale^7^, ^42^. The 95% confidence interval (CI) of the molecular dating is shown as green bars on selected nodes. The presence (solid circles) and absence (empty circles) of DsrA and DsrB in archaeal genomes are shown. DsrA and/or DsrB in specific archaeal lineages are also shown by coloring respective branches. The colors of the RA‐DsrAB subclades are consistent with Figure 1. The transfer events of DsrAB obtained by reconciling the species tree and the DsrAB tree are shown as brown dots on the respective branches. The numbers in brown represent the time scale of the branches used as constraints for tree calibration in Figure 4. The name of each genome contains the classification and the NCBI accession number. This classification is derived from the GTDB taxonomic ranking, where “c_”, “o_”, “f_”, and “g_” represent the classifications of class, order, family, and genus, respectively, and are used before the name of a candidate ranking. The habitats from which the genomes were obtained, as well as the temperature of the habitats, are shown in colored boxes.

**Figure 3:**
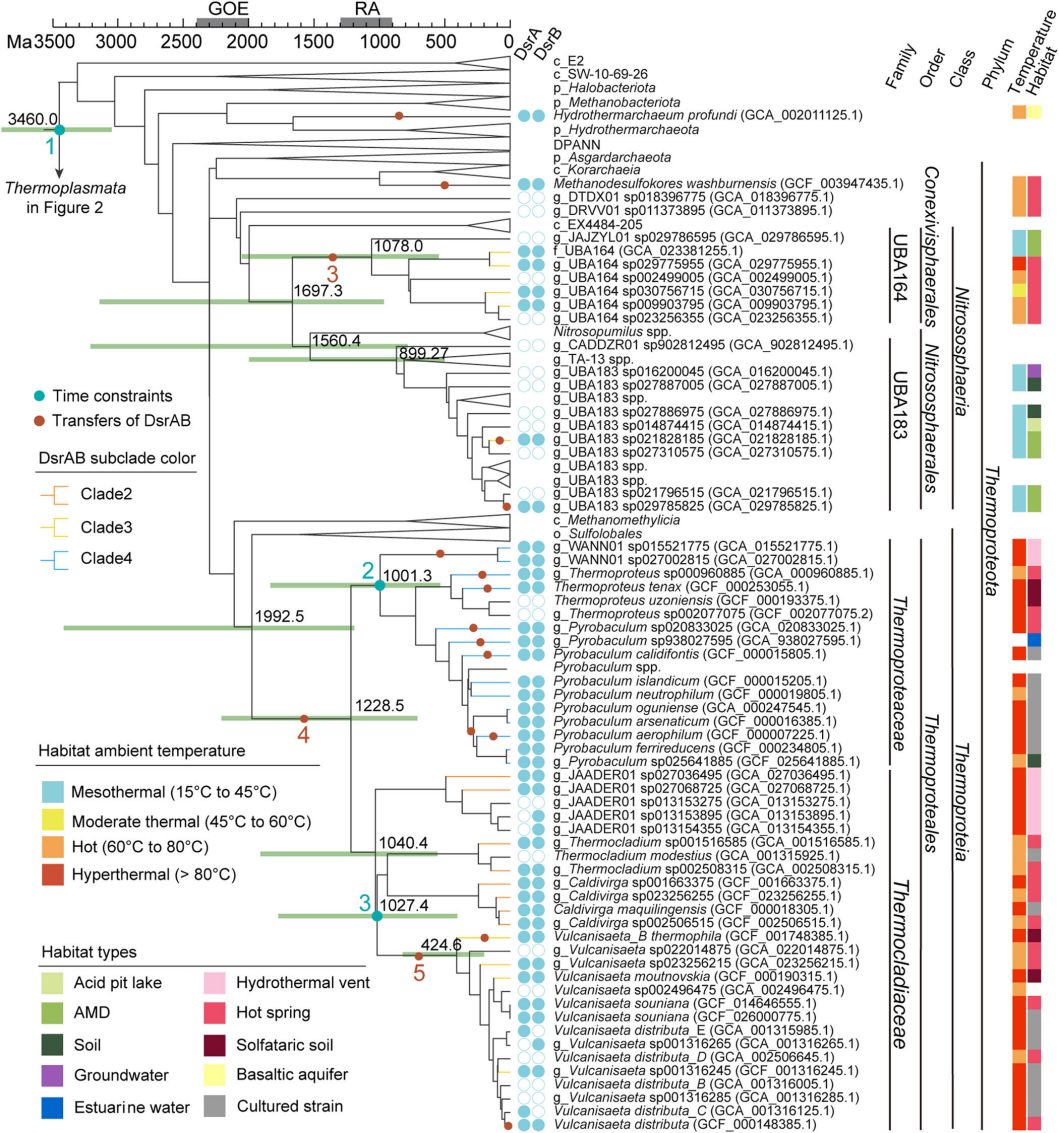
Evolutionary time scales and habitat feature of *Thermoproteota* encoding RA‐DsrAB. Only the *Thermoproteota* clade of the tree described in Figure 2 is shown here for typeset.

The reconstruction of the evolutionary history of RA‐DsrAB proteins in the tree of archaea shows that the genes of these proteins were frequently transferred within and between the lineages of two archaeal phyla: *Thermoplasmatota* and *Thermoproteota* (Figures 2 and 3). Specifically, RA‐DsrAB proteins of clade 1 and clade 5 were transferred within the UBA184, ARK‐15, GCA‐001856825, and *Thermoplasmataceae* family‐level clades of the phylum *Thermoplasmatota* (Figure 2). Homologs of clade 3 were transferred to the *Thermocladiaceae*, UBA164, and UBA183 family‐level clades of the phylum *Thermoproteota* (Figure 3). Homologs of clade 2 were transferred to *Thermocladiaceae* of *Thermoproteota*, and homologs of clade 4 were transferred to *Thermoproteaceae* of *Thermoproteota* (Figure 3).

### Dating the evolutionary origin and diversification of basal‐type and reductive archaeal‐type DsrA

First, molecular dating was conducted on a species tree comprising representative archaeal families and additional archaeal taxa encoding DsrAB. Four nodes were set as time constraints for calibration (Figures 2 and 3). The root of archaea was dated to 3.460 Ga. The detectable transfers of RA‐DsrAB to these taxa occurred as early as 1.993 Ga and as late as recently.

**Figure 4:**
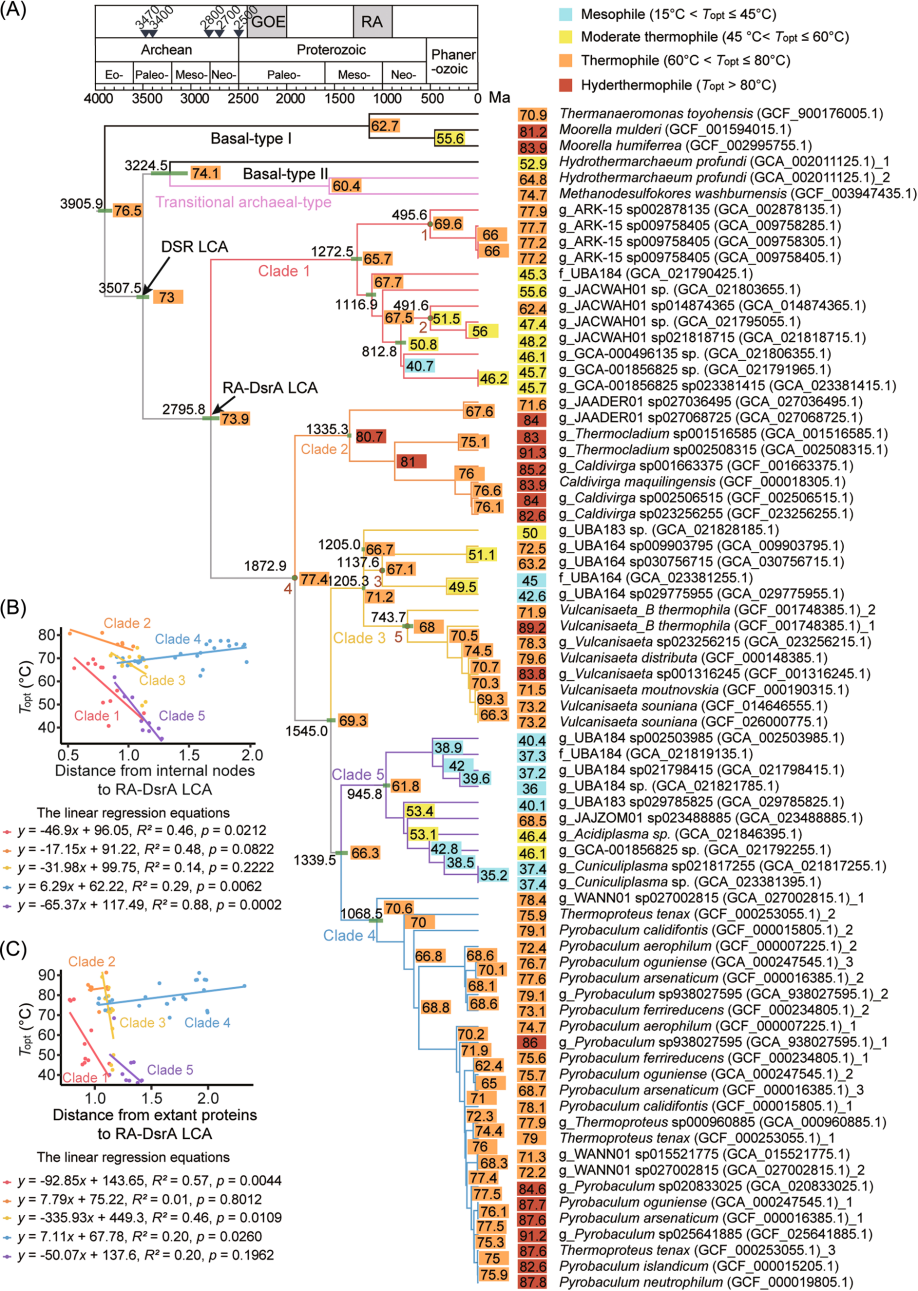
Molecular dating of the basal‐type DsrA and RA‐DsrA. (A) The maximum likelihood phylogenetic tree (model automatically selected as LG + I + G4) reconstructed with representative DsrA and DsrB homologs from 74 DsrAB sequences with 289 amino acid sites. The DsrB clade was set as the outgroup (not shown here) and only the DsrA part of the tree is shown. The constraints of nodes in brown are transferred from Figures 2 and 3 (Table S4). Dating values on selected nodes are displayed with 95% CI values, which are shown as green bars. The colors of the basal‐type DsrA, the transitional archaeal‐type DsrA, and the RA‐DsrA subclades are consistent with Figure 1. The period of the RA and the GOE are illustrated in the geological timeline^7^, ^42^. Geological periods are marked as Ecoarchean, Paleoarchean, Mesoarchean, Neoarchean, Paleoproterozoic, Mesoproterozoic, and Neoproterozoic. The triangle represents the geological evidence of microbial sulfate reduction in Archean (Table S10)^8^, ^43^, ^44^, ^45^, ^46^, ^47^, ^48^. The *T*_opt_ values of the ancestral and extant protein sequences are shown at the internal nodes and the external nodes, respectively. Color shades indicate four temperature ranges. (B, C) Correlation of evolutionary distance and *T*_opt_ in internode ancestral (B) or extant (C) DsrA proteins. Linear regression equations are shown.

The molecular dating results of the archaeal species tree were used to calibrate the DsrA tree. Specifically, five branches of the archaeal species tree containing major DsrAB transfer events (Figures 2 and 3) were selected based on the results of ALE and projected onto five nodes in the DsrA tree (Figure 4A). The time scales of the branches in the archaeal species tree were set as the time scales of the respective nodes in the DsrA tree. These five nodes and the root with an upper age of 4.38 Ga in the DsrA tree were used as constraints to calibrate the tree time scale (Table S4). The five constraint nodes include (1) the LCA of homologs encoded by g_ARK‐15 archaea (1.078 Ga–465.8 Ma), (2) the LCA of homologs encoded by three g_JACWAH01 archaea (716.6–465.6 Ma), (3) the LCA of homologs encoded by four f_UBA184 archaea (1.697–1.078 Ga), (4) the LCA of homologs of *Thermoproteales* (1.993–1.229 Ga), and (5) the LCA of homologs of *Vulcanisaeta* (1.027 Ga–424.6 Ma) (Figures 2, 3 and 4A).

Molecular dating results show that the root of DsrA dates back to 3.906 Ga in the Eoarchean Era (Figure 4A). The LCA of the basal type II DsrA and the RA‐DsrA, which is considered the LCA of DSR, was dated to 3.507 Ga in the early Paleoarchean Era, a time just before the two oldest geological records of the DSR (3.470 and 3.400 Ga)^43^, ^44^ (Figure 4A). Moreover, the LCA of extant RA‐DsrA was dated to 2.796 Ga at the Meso–Neoarchean boundary, prior to the GOE (2.5–2.4 to 2.1–2.0 Ga)^7^, ^49^, ^50^, ^51^. The diversification of RA‐DsrA subclades occurred after this point, and each clade began to diverge from 1.335 Ga (clade 2) to 945.8 Ma (clade 5). This period spans from the mid‐Mesoproterozoic to the early Neoproterozoic, and coincides with the Rodinia Assembly (RA, 1.3 Ga to 900 Ma)^42^, ^52^ (Figure 4A).

### The environmental temperature at the origin of DSR

The amino acid composition and secondary structure of microbial proteins are often optimized through evolution to adapt to their habitat temperature^53^, ^54^. To track the thermal adaptation of DsrA throughout its evolutionary history, we predicted the potential *T*_opt_ values (values considering both the protein sequence and structure) for both extant and ancestral DsrA sequences (Figure 4A and Table S5)^55^.

The root of the DsrA tree had a *T*_opt_ value of 76.5°C (Figure 4A). The *T*_opt_ of the LCA protein of DSR was 73°C, while the LCA proteins of basal type II DsrA and transitional archaeal‐type DsrA had a *T*_opt_ of 74.1°C. The LCA of RA‐DsrA had a *T*_opt_ value of 73.9°C. These results suggest that early versions of DsrA, including the one at the origin of the functional Dsr cascade in DSR, were likely adapted to hot environments.

### The environmental adaptation history of RA‐DsrAB‐encoding archaea

While the LCA of the extant RA‐DsrA was likely adapted to a hot environment (73.9°C), copies of the RA‐DsrA clades were then frequently transferred between lineages of *Thermoproteota* and *Thermoplasmatota* in environments of various levels of ambient temperatures (Figure 4A). By calculating *T*_opt_ of the ancestral protein sequences, we found that the evolutionary diversification of RA‐DsrA followed an overall tendency from hyperthermal to mesothermal environments after its origin. However, this overall tendency varied among RA‐DsrA subclades. The *T*_opt_ values of the ancestral proteins and the evolutionary distance of their nodes to the LCA of RA‐DsrA were negatively correlated in clades 1, 2, and 5 (*R* ^*2*^ > 0.46, *p *< 0.1, Figure 4B), but weakly correlated in clade 3 (*R* ^2^ = 0.14, *p* = 0.2222), indicating that these DsrA proteins originally in hyperthermal and hot environments adapted to moderate thermal and mesothermal environments during the evolutionary process. In contrast, a positive correlation was found between *T*_opt_ values and evolutionary distance in the DsrA ancestral proteins of clade 4 (*R* ^2^ = 0.29, *p* = 0.0062), suggesting that these proteins adapted to environments of even higher temperatures than their thermophilic ancestors (i.e., proteins adapted to environments with ambient temperature from 60°C to 80°C) during later evolution.

The extant genes of RA‐DsrA are encoded by archaea from habitats with different temperature and pH levels (Figure 2 and Figure 3). A similar correlation tendency was observed between *T*_opt_ values and evolutionary distance to the LCA in extant RA‐DsrA proteins, but in clade 2, a weak positive correlation was observed (Figure 4C).

## DISCUSSION

The time, environment, and molecular basis of the origin of DSR remain long‐standing scientific questions. Isotopic evidence suggests that DSR activities date back to Archean, with the oldest record being 3.47 Ga^8^. The DSR pathway, which is catalyzed by Dsr proteins, is considered to be among the oldest and most prevalent in microorganisms^15^, ^16^. As *dsr* genes are transferred extensively between bacterial and archaeal lineages, tracing the origin of the functional Dsr protein assemblies and their microbial hosts is challenging. Although many studies have conducted phylogenetic analyses of Dsr proteins, including the key catalytic enzymes DsrAB, only one recent study has explored the assembly of Dsr proteins in the early stages of evolution^28^. According to this study, archaea may have been the first to develop the complete set of the DsrABCMK(N) complex required for sulfite reduction. By recruiting genomes encoding Dsr proteins from the updated public database, specifically metagenomes sampled in mesothermal environments, we recovered further details of the basal and reductive archaeal types of DsrAB and their co‐assembled Dsr proteins. Using this updated information, we discovered that the functional Dsr assembly for DSR, including essential components DsrABCNM, likely happened before the LCA of basal type II DsrAB, RA‐DsrAB, and transitional archaeal‐type DsrAB, with an age of 3.51 Ga (Figure 1 and Figure 4A). This timing is highly coherent with the oldest known geological record of DSR (3.47 Ga)^8^. Therefore, our results provide the most congruent evidence to date on the enzymatic basis of DSR and suggest that the original DSR was catalyzed by a minimum enzyme cascade comprising DsrABCNM in the Paleoarchean Era. The time of origin of DsrK remains unresolved due to limited data. It may have been present at the onset of DSR (3.51 Ga). Otherwise, it could have emerged later in the common ancestor of the RA‐DsrAB lineages and subsequently been transferred to bacteria containing reductive bacterial‐type DsrAB (Figure 1). The DsrK subunit, which harbors a [4Fe–4S] cluster, plays a crucial role in DSR by catalyzing the reduction of the DsrC‐trisulfide^29^. In the absence of DsrK, DsrM is likely incapable of supporting complete DsrC reduction. Nevertheless, DsrM may still function as a membrane‐bound electron transfer module, potentially interacting with alternative redox partners^30^.

Our result suggests that the host microorganism encoding DsrAB can be either a bacterium or an archaeon at the origin of DSR. Before the node of DSR LCA, all the branches were of DsrAB encoded by bacteria (Figure 1). After this node, one branch contained the basal type II DsrAB including bacterial sequences exclusively, except for one archaeal copy. The other branch comprised two purely archaeal clusters: RA‐DsrAB and the transitional archaeal‐type DsrAB. Due to the current limitations in data availability, it is difficult to determine if DsrAB was transferred horizontally from a bacterial donor to a bacterial or archaeal receptor at the DSR LCA node.

Compared to the extant DsrA in hyperthermal to mesothermal environments, the *T*_opt_ of the predicted ancestral DsrA protein at the origin of DSR (73°C) suggested that the microbial host might live in or very close to the hot spring vents (Figures 2, 3, 4). However, it is worth noting that enzymes with a high *T*_opt_ may remain functional in microorganisms living in a slightly lower ambient temperature (Figures 2 and 3). This observation can readily explain the estimated ambient temperature during the formation of the earliest geological records of DSR ( < 60°C)^8^. Although this finding corroborates the conclusion of a previous genomic study to some extent^17^, the unresolved (bootstrap <90) branch from mesothermal environments in the DsrAB tree of that study made its conclusion arguable. Moreover, the presence of *dsrAB* branching as basal clades encoded by extant thermophiles and hyperthermophiles (i.e., organisms that thrive at ambient temperature >80°C), as shown in that study, does not necessarily imply that DSR originated in hot and hyperthermal environments. Dsr genes were frequently transferred between bacteria and archaea. The copies of extant thermophilic and hyperthermophilic archaea may have been transferred from a donor archaea or bacteria that once lived in mesothermal and moderate thermal environments. Therefore, the temperature at the origin of Dsr cannot be addressed by directly referring to the habitat temperature of extant *dsrA/dsrB*‐carrying microorganisms. In this study, we applied an ancestral sequence analysis of extant DsrAB proteins to determine the origin of DSR. This approach recruited phylogenetic models to infer the ancestral protein sequences independently of the host microorganisms that encode them. The *T*_opt_ values inferred from these ancestral sequences thus reflect the intrinsic features of the enzymes themselves in temperature adaptation.

While DSR probably emerged before the LCA of RA‐DsrAB, RA‐DsrAB is one of the most well‐adapted and diverse DsrAB types and remains the most ancient type for which there is experimental evidence of DSR activity. By the time of LCA of RA‐DsrAB, which is dated to 2.795 Ga, a minimum set of the DsrABCNMK cascade had been established and was generally preserved across its five subclades (Figure 1). After their evolutionary origins in hot environments, some RA‐DsrAB‐carrying archaea gradually adapted to mesothermal and moderate thermal environments, while others stayed in hot habitats or adapted to hyperthermal environments. During the Mesoarchean Era and the Neoarchean Era (3.2–2.5 Ga), the Earth's atmosphere began to accumulate free oxygen, likely driven by oxygenic photosynthesis, until the atmosphere was permanently oxidized in the GOE^7^, ^56^. Throughout this gradual oxidation process, weathered sulfides were imported into diverse environments on a larger scale. The widespread availability of sulfur oxides under varied physicochemical conditions created numerous ecological niches for sulfite‐reducing archaea^2^, ^6^, ^57^, ^58^, supporting an adaptive expansion of both RA‐DsrAB (Figure 4) and their archaeal hosts (Figures 2 and 3) after the GOE. In particular, during the formation of the early Neoproterozoic supercontinent Rodinia (1.3–0.9 Ga)^42^, ^52^, ^59^, increased orogenic events might have led to the exposure of sulfide mineral deposits^60^, which were oxidized to produce sulfuric acid and contributed to localized acidic environments. These conditions may have facilitated the emergence of AMD‐like environments and consequently the thriving of mesophiles and acidophiles encoding RA‐DsrAB^61^, ^62^, ^63^. Indeed, RA‐DsrAB in clades 1, 3, and 5 were transferred to the phyla *Thermoplasmatota* and *Nitrososphaeria* in mesothermal acidic environments between 1.340 and 1.205 Ga (Figures 2, 3, 4).

In summary, we reconstructed the phylogenetic trees of DsrAB and other Dsr proteins, and inferred that the core components DsrABCNM of the DSR pathway were already assembled in the Paleoarchean Era. Molecular dating of both species and protein trees alongside ancestral sequence reconstruction indicates that the DSR pathway likely originated at around 3.508 Ga with an estimated ancestral DsrA *T*_opt_ of 73°C, supporting the hot origin hypothesis of DSR. Additionally, our analysis of RA‐DsrAB subclades revealed how enzymes and species have adapted to diverse geological environments over time. This work expands our understanding of the early evolutionary assembly of the DSR, but several questions remain open. The scarcity of basal‐type genomes limits the analytical resolution of the earliest steps in the Dsr cascade assembly process, leaving the archaeal versus bacterial origin unresolved. Future recovery of deep‐branching genomes, coupled with functional assays and tighter geochemical constraints, will be essential to refine our understanding of microbial DSR origin and early diversification.

## MATERIALS AND METHODS

### DsrA and DsrB homolog search

3,031 AMD MAGs and 65,703 GTDB representative genomes (207_v2)^28^ were collected to search for DsrA and DsrB homologs. The AMD MAGs were obtained from data deposits of our two previous studies (NCBI BioProjects PRJNA666025 and PRJNA666095)^37^, ^38^. 90 AMD sediment samples were collected from 18 mine sites across six provinces in southern China between August and October 2017,^37^ and two tailings cores were collected from the Fankou Pb/Zn sulfidic mine tailings site in Shaoguan, Guangdong, China, in October 2017 (Figure S12A)^38^. The detailed DNA extraction procedure and physicochemical parameter assay methods can be found in two previous studies. Coding proteins were predicted from the AMD MAGs using Prodigal v2.6.3 with the following parameters: “‐p meta ‐g 11 ‐f gff ‐q –m”^64^. Taxonomic classification of these MAGs was performed using GTDB‐tk 207_v2^65^. The coding proteins and taxonomic classification of the GTDB representative genomes were obtained from the GTDB website. The hidden Markov models of DsrA and DsrB from the KOfam database (downloaded on July 4, 2024)^66^ and the TIGRfam database (downloaded on July 10, 2024)^67^ databases were used to search for DsrA and DsrB homologs using hmmscan v3.2.1 with the “‐T 223” option set for DsrA and the “‐T 205” option set for DsrB (K11180 for DsrA and K11181 for DsrB, TIGR02064 for DsrA, and TIGR02066 for DsrB)^15^, ^68^, ^69^. Only *dsrA* and *dsrB* gene pairs that were adjacent to each other within the same genome were retained, and *dsrA* and *dsrB* gene sequences of the same pair were concatenated for downstream analysis (*dsrAB* is used to refer to the concatenated *dsrA* and *dsrB* genes). We used CD‐HIT v4.6.8^70^ to remove redundant sequences with the parameter set “‐c 0.75 ‐n 5.” Further verification for DsrA sequences was conducted based on the presence of both the conserved siroheme‐binding Cx5CXnCX3C motif and the [Fe_4_S_4_] cluster‐binding CX2CX2C motif, resulting in the retrieval of 415 concatenated DsrAB sequences^17^. Multiple DsrAB copies within each genome were flagged (Table S1). Annotations of other Dsr proteins were conducted using Disco^71^ and SCycDB^72^. The CbiA, CobB, and CfbB sequences were selected according to a previous study for analyzing the phylogeny of DsrN^28^.

### Phylogenetic trees of Dsr proteins

The multiple sequence alignment of DsrAB, DsrC, DsrN, DsrM, DsrK, DsrJ, DsrO, DsrP, DsrD, DsrT, and DsrEFH proteins was constructed using MAFFT v7.505 with default parameters^73^. Gaps were removed using TrimAl v1.4.rev15 (“‐gt 0.95 ‐cons 50”)^74^. The maximum likelihood tree was reconstructed using IQTree v.2.2.0.3 (“‐m MFP ‐B 1000 –bnni”)^75^ and visualized in iTOL v7^76^. Treemmer v0.3 (‐RTL 0.3)^77^ was then used to reduce the number of branches in the trees of reductive bacterial‐type DsrAB and oxidative bacterial‐type DsrAB to 31. Paralogous rooting was conducted as suggested in a previous study to determine the root of this DsrAB concatenated tree^17^. This involved the pooling of paired DsrA and DsrB sequences that were not concatenated, followed by the construction of a phylogenetic tree using the same method as that for the concatenated DsrAB tree. Paralogous rooting was performed using an equivalent number of DsrA and DsrB subunits from the concatenated DsrAB tree. The monophyletic clade, comprising three *Chloroflexota* and eight *Firmicutes* sequences (sequences from the basal type I DsrAB), emerged as the most basal group for both the DsrA and the DsrB branches, consistent with previous studies (Figure S13)^11^, ^41^. Therefore, we used these 11 taxa as the outgroup to root the DsrAB concatenation tree. The DsrC tree was rooted with the basal and archaeal clusters as counterparts of the DsrAB tree (Figure S2). CbiA, CobB, and CfbB sequences were used as outgroups to root the DsrN tree (Figure S3). DsrE, DsrF, and DsrH trees were also rooted using the paralogous rooting method (Figure S11). Other phylogenies were rooted at their midpoints.

### Ancestral sequence generation and feature prediction

Amino acid sequences at internal nodes of the DsrA phylogenetic tree were computationally inferred from the sequence alignment and tree topology using the codelml program in the PAML v4.9 package^78^. The Bayesian statistical framework, incorporating a Gamma distribution, was used to infer the posterior amino acid probability per site. No molecular clock was set during this analysis. The universal code (icode = 0) and the fixed branch length option (Mgene = 0) were used. *T*_opt_ of ancestral and extant DsrAB sequences were predicted using DeepET^55^.

### Phylogenetic inference of the species tree

A phylogenetic species tree of representative archaeal and bacterial lineages encoding RA‐*dsrAB* was reconstructed. Specifically, the quality of 12 nonredundant RA‐*dsrAB*‐encoding AMD MAGs (CD‐HIT “‐c 0.75 ‐n 5”) was assessed using CheckM v1.1.3^79^. Those with completeness >75% and contamination <5% were included (Table S6). For GTDB genomes (r220), those with completeness >75% and contamination <5% were selected for downstream analysis. Two GTDB‐obtained genomes with the highest‐quality values, calculated as “completeness – 4 × contamination” as suggested in a previous study^80^, were selected as representatives of each archaeal phylum (except for the phyla *Thermoplasmatota* and *Thermoproteota*, in which two representative genomes were selected per class). Family‐level GTDB‐obtained genomes carrying *dsrAB* in *Thermoplasmatota* and *Thermoproteota* were further selected. Four bacterial genomes were randomly selected and downloaded from the GTDB website as outgroup species. Finally, 306 high‐quality genomes were selected for downstream analysis. Orthologs were identified using OMA v2.6.0^81^, and 47 candidate marker genes were retained based on their presence in 261 (85%) of the 306 high‐quality genomes. The marker genes were annotated using the NCBI BLAST website, and the best hit for each marker gene is shown in Table S7. The multiple sequence alignments of these candidate individual gene trees were generated using MAFFT with default parameters, followed by removal of the alignment gaps using TrimAl (“‐gappyout”). All the candidate marker genes were included after using the scripts tre_make_split.pl and tre_discordance_two.pl provided in a previous study to confirm the topological concordance between individual gene trees^82^. Multiple sequence alignments of individual marker genes were concatenated using catfasta2phyml.pl (https://github.com/nylander/catfasta2phyml). Compositional heterozygous sites of the concatenated marker genes (0%, 5%, 10%, 15%, 20%, 25%, 30%, 35%, and 40%) were removed using the script alignment_pruner.pl, which was provided in a previous study^83^. Species trees were then inferred using IQTree with the parameters “‐m LG + PMSF + F + G ‐B 1000 –bnni.”

### Molecular dating of the archaeal species tree and the DsrA tree

The node divergence times of the archaeal species tree were estimated in MEGA v11.0.13 using the RelTime‐ML and the LG + G models^84^. The root of archaea (4.38–3.46 Ga)^85^ and three nodes related to the GOE (i.e., the root of *Thermoproteales, Thermoproteaceae*, and *Thermocladiaceae* (except g__JAADER01), <2.32 Ga)^86^, ^87^ were selected as calibration constraints (Table S4). To determine the latter three, we used hmmsearch v3.2.1 with the option “domE 1‐e5” to search for the cytochrome oxidases (K02274 and K02275) and cytochrome bd quinol oxidases (K00425 and K00426) in aerobic archaea and identify clades with anaerobic ancestors (Figure S14 and Table S8). These taxa would have evolved from anaerobic to aerobic lifestyles after the rise of atmospheric oxygen 2.32 Ga^87^.

For the molecular dating of the DsrA tree, DsrA and DsrB in 306 genomes were searched using hmmscan v3.2.1 with the Hidden Markov Models in the KOfam (K11180 for DsrA and K11181 for DsrB)^66^ and TIGRfam (TIGR02064 for DsrA and TIGR02066 for DsrB)^67^ databases, with the option “‐T 223” for DsrA and “‐T 205” for DsrB, respectively^15^, ^68^, ^69^. Neighboring DsrA and DsrB sequences within the same genome were retained, and an equivalent number of representative DsrA and DsrB sequences were obtained for multiple sequence alignment using MAFFT with default parameters^73^. Alignment gaps were removed using TrimAl (“‐gt 0.95 ‐cons 50”)^74^, and the maximum likelihood tree was reconstructed using IQTree (“‐m MFP ‐B 1000 –bnni”)^75^. The DsrB clade was used as the outgroup for rooting. The results of the species tree dating were used as constraints for the DsrA tree dating (Table S4). A DsrAB concatenated tree was reconstructed following the same steps as those for the DsrA tree, but with concatenated DsrA and DsrB sequences in the multiple sequence alignment.

The reconciliation of the DsrAB gene tree with the species tree was conducted using ALE v1.0 (https://github.com/ssolo/ALE), and the outputs with frequency of transfer events less than 0.3 were filtered out (Table S9). By screening the branch where DsrAB transfer events were detected, the time range of the branch on the species tree was set as the constraint for the LCA node of the corresponding clade on the DsrA tree. Only clades that contained three or more branches were used as constraints. The tree files were visualized using iTOL^76^ and tvBOT v2.6.1^88^.

### Gene abundance calculation of *dsrAB* clades in AMD metagenomes

Pairwise sequence alignments of the DsrA proteins in the DsrAB tree were performed using Diamond blastp^89^ with the parameter “‐‐more‐sensitive ‐‐max‐target‐seqs. 60.” Heatmaps of pairwise sequence identities were then plotted to assess the identity thresholds between the five DsrA clades (Figure S1). An identity threshold of 60 was chosen to discriminate between DsrA proteins of different clades.

Quality control of the raw reads from the AMD sediments was conducted as previously described^37^, and high‐quality reads were assembled into contigs using SPAdes v3.14.1^90^. The abundance of the contigs in the AMD samples was calculated using CoverM^91^ with minimap2‐sr. The coverage method was trimmed_mean, which calculates the average number of aligned reads overlapping each position after removing the deepest and shallowest covered positions^91^. The DsrA proteins in the DsrAB tree were searched against the coding proteins of the contigs using Diamond blastp with the parameter “‐k 5 ‐e 1e‐5 ‐‐id 60.” The abundance of the resulting DsrA proteins was defined as the abundance of the genomic contigs encoding them.

### Statistical analyses

All statistical analyses were performed in the R environment (v.4.3.2) using R packages. Specifically, Spearman correlation analyses were performed using the “corrplot” function in the corrplot package (v 0.92)^92^ to assess the relationships between abundances of *dsrAB*‐carrying archaeal populations and physicochemical factors in 90 AMD sediment samples. Redundancy analysis (RDA) was performed to show the community structure of *dsrAB*‐carrying archaea in response to physicochemical factors using the vegan package (v 2.6‐4)^93^. Prior to RDA, the microbial abundance matrix was standardized using the Hellinger method with the “decostand” function, and the environmental factor matrix was standardized by taking logarithms. The “envfit” function, which fits environmental factors to species distributions, was used to screen environmental factors in RDA. Environmental factors with *p* < 0.001 were retained. The Monte Carlo permutation test with the “permutest” function was used to evaluate the RDA results (*p* < 0.001). Nonparametric multivariate analysis of variance (Adonis) was used to test the abundances of *dsrAB*‐carrying archaea in different mineral types containing >10 samples (i.e., Cu, Pb/Zn, and Polymetallic) using vegan. The distance between the internal node or branch and the LCA of the RA‐DsrAB was calculated using the “dist. node” function of ggpmisc (v 0.6.0)^94^. For each of the five clades, linear regressions were performed between the *T*_opt_ values predicted for the ancestral proteins of the internal nodes or the extant proteins of each branch, and the evolutionary distance of each internal node or branch to the last common ancestor. The lm function was used to calculate the equations of the linear regression curves, and to extract the *R* ^2^ and *p* values. Data visualization was performed using the ggplot2 package (v3.3.4) and the gglayer package (v0.0.4)^95^, ^96^. We used analysis of variance (ANOVA) to analyze the differences in the abundance of the five RA‐DsrAB clades among different mineral types or sampling sites.

## AUTHOR CONTRIBUTIONS

**Lingyun Tang**: Data curation; formal analysis; investigation; methodology; visualization; writing—original draft. **Zhenhao Luo**: Data curation; funding acquisition; methodology. **Shaoming Gao**: Writing—review and editing. **Zhiliang Lin**: Data curation; methodology. **Mengqi Sun**: Funding acquisition; writing—review and editing. **Runsheng Li**: Writing—review and editing. **Shu‐Hong Gao**: Data curation; methodology. **Geng Wu**: Writing—review and editing. **Yiliang Li**: Writing—review and editing. **Linan Huang**: Conceptualization; funding acquisition; writing—review and editing. **Lu Fan**: Conceptualization; funding acquisition; investigation; project administration; supervision; writing—review and editing.

## ETHICS STATEMENT

The authors have nothing to report.

## CONFLICT OF INTERESTS

The authors declare no conflict of interests.

## Supporting information

Supplementary Information.

Supplementary Table.

## Data Availability

The tree files and the nexus files of tree dating can be found at Figshare (https://doi.org/10.6084/m9.figshare.30351535).
