# Supplementary material for: A hot origin of dissimilatory sulfite reduction catalyzed by DsrAB in the Paleoarchean Era
Source: mLife. 2026 Feb 23;5(1):108–21. doi: 10.1002/mlf2.70066 (PMC12948486; doi:10.1002/mlf2.70066)
Supplement: Supplementary file 1 — Supplementary Information. [file MLF2-5-108-s002.pdf]

Supplementary Information

The PDF file includes:

Supplementary Text

Figure S1 to S16

Other Supplementary Materials for this manuscript include the following:

Table S1 to S15

Supplementary Text

dsrA/dsrB-carrying archaea in AMD environments

In the 92 AMD samples collected in our previous studies [1, 2], we identified 31 metagenome assembled genomes (MAGs) of completeness > 50% and contamination < 10% belonging to archaeal phyla *Thermoplasmatota* (28) and *Thermoproteota* (3) that contained *dsrA* and/or *dsrB* genes (Figure S12A, Table S11). *dsrA/dsrB*-carrying *Thermoplasmatota* MAGs belonged to three family-level groups of the class *Thermoplasmata*, namely GCA-001856825, *Thermoplasmataceae,* and UBA184, whereas *dsrA/dsrB*-carrying *Thermoproteota* MAGs belonged to two family-level groups of the class *Nitrososphaeria*, including UBA164 and UBA183 (Figure S12B, Table S11).

The abundances of *dsrA/dsrB*-carrying archaeal families showed significant variability within the same or across different mineral types (Figure S12B). *Thermoplasmataceae* was the dominant family in the Fe_3O_4, polymetallic, and Sn/Zn mines and was widely distributed across almost all mineral types. UBA164 was dominant in Cu/Fe and Pb/Zn mines but was absent or in low abundance in other mines (Figure S12B).

The abundance and composition of *dsrA/dsrB*-carrying archaea were correlated with specific physicochemical parameters in AMD samples from different mine types. The total abundance of *dsrA/dsrB*-carrying archaea correlated positively with electrical conductivity (EC), Pb, Cu, Zn, and Mn concentrations, respectively (Spearman r > 0.3, $P \leqslant 0.003$), and negatively with ferric (Spearman r = -0.379, P = 0.000227) in all

AMD samples (Table S12). Redundancy analysis (RDA) shows that the concentrations of Pb, Fe, Mn, Cu, and Zn were correlated with the community structure of *dsrA/dsrB*-carrying archaea in all AMD samples (all Q values = 0.0028 after Benjamini-Hochberg correction of the P values) (Figure S12C, Table S13).

The composition of *dsrA/dsrB*-carrying archaeal MAGs showed a significant separation by mineral type among all AMD samples (Adonis $R^2 = 0.337$, $P = 0.001$) (Table S14). The community structure of *dsrA/dsrB*-carrying archaea in Sb and some polymetallic mine samples was distinguished from other samples based on the concentration of heavy metals (Figure S12C). For mineral types containing more than 10 samples (including polymetallic mines, Pb/Zn mines, and Cu mines), observing the correlation between the abiotic factors and the abundance of *dsrA/dsrB*-carrying archaea is beneficial. In polymetallic mines, the abundance of *dsrA/dsrB*-carrying archaea negatively correlated with ferric and sulfate (Spearman r $\leqslant$ -0.42, $P < 0.02$), and positively correlated with EC and TOC (Spearman r > 0.6, $P < 0.0002$) (Table S12). In Pb/Zn mines, the abundance of *dsrA/dsrB*-carrying archaea was positively correlated with ferrous and sulfate (Spearman r > 0.7, $P < 0.00065$) (Table S12). In contrast, the abundance of *dsrA/dsrB*-carrying archaea in Cu mines exhibited weak correlations with the measured abiotic factors, and the associated Spearman tests were not statistically significant (all Spearman $P > 0.05$, Table S12).

Environmental distribution of RA-DsrAB clades in AMD

The abundance of the RA-DsrAB from different clades in AMD sediments of various mineral types may provide insights into their specific environmental adaptations.

Heterogeneity in the abundance of the five RA-DsrAB clades was observed between both mineral types (ANOVA test, P = 0.00105, Table S15) and sampling sites (ANOVA test, P = 0.00454, Table S15). Clade 2 RA-DsrAB proteins were the most abundant in FeS_2, Sn/Zn, Pb/Zn, Polymetallic, and Sb mines, with considerable variation in their abundance across different mineral types (Figure S15, Table S15). In Fe_3O_4 mines, the RA-DsrAB proteins of clade 1 were the most abundant. In contrast, the RA-DsrAB proteins of clade 3 were the most abundant in Cu and Cu/Fe mines and were absent in the Fe_3O_4 mines. The RA-DsrAB proteins of clade 4 were generally present at low abundance in all mineral types and were absent in FeS_2 mines. Within a given mineral type, the abundance of RA-DsrAB proteins varied between sampling sites. For example, in the Pb/Zn mines, RA-DsrAB from clade 2 had the highest abundance in HSP and YS, whereas RA-DsrAB from clade 3 and clade 5 had the highest abundance in FK and SKS (Figure S16). This pattern was also present in Cu and polymetallic mines.

The abundance of RA-DsrAB clades was correlated with different physicochemical factors across mineral types, indicating potentially complex interactions between RA-DsrAB functions and the AMD environment. In all samples, the abundance of clade 5 showed a positive correlation with Mn (R^2 = 0.28, P < 0.05) (Figure S16). In Pb/Zn mines, the abundance of clade 3 was positively correlated with ferrous (R^2 = 0.75, P < 0.001), sulfate (R^2 = 0.85, P < 0.001) and Pb (R^2 = 0.64, P < 0.05) (Figure S17), and the abundance of clade 1 was positively correlated with Pb (R^2 = 0.77, P < 0.001). In polymetallic mines, the abundance of clade 1 was positively correlated with

pH and Fe (R^2 = 0.58 and 0.61, P < 0.001) (Figure S16).

Although RA-DsrAB-carrying archaea have been previously detected in mesothermal acidic environments, including acid pit lakes and peat soil [3, 4], the extensive sampling effort in this study enabled the investigation of the relationship between environmental factors and RA-DsrAB-carrying archaea. The findings revealed a positive correlation between the abundance of RA-DsrAB-carrying archaea and physicochemical factors, including EC, the concentrations of Pb, Cu, and Mn (Table S12). The increased EC suggests a higher concentration of ions, which indicates an enhanced mining process leading to the leaching of sulfur oxides and heavy metal ions (e.g., Pb, Cu, and Mn ions) into the environment. The potentially heightened level of sulfur oxides may have fostered the proliferation of sulfite-reducing archaea; however, the assessment of sulfite concentration remains deficient in this study. More detailed data on environmental factors is expected in future studies to enhance our comprehension of the pivotal factors that propel adaptive diversification of RA-DsrAB-carrying archaea in AMD.

Reference

1. Gao S, Paez-Espino D, Li J, Ai H, et al. Patterns and ecological drivers of viral communities in acid mine drainage sediments across Southern China. *Nature Communications* 2022; **13**: 2389. https://doi.org/10.1038/s41467-022-30049-5
2. Luo Z, Li Q, Chen N, Tang L, et al. Genome-resolved metagenomics reveals depth-related patterns of microbial community structure and functions in a highly

stratified, AMD overlaying mine tailings. *Journal of Hazardous Materials* 2023; **447**: 130774. https://doi.org/10.1016/j.jhazmat.2023.130774

3. Ayala-Muñoz D, Burgos WD, Sánchez-España J, Couradeau E, Falagán C, Macalady JL. Metagenomic and Metatranscriptomic Study of Microbial Metal Resistance in an Acidic Pit Lake. *Microorganisms* 2020; **8**: 1350. https://doi.org/10.3390/microorganisms8091350
4. Lin X, Handley KM, Gilbert JA, Kostka JE. Metabolic potential of fatty acid oxidation and anaerobic respiration by abundant members of *Thaumarchaeota* and *Thermoplasmata* in deep anoxic peat. *The ISME Journal* 2015; **9**: 2740–2744. https://doi.org/10.1038/ismej.2015.77

Supplementary Figures

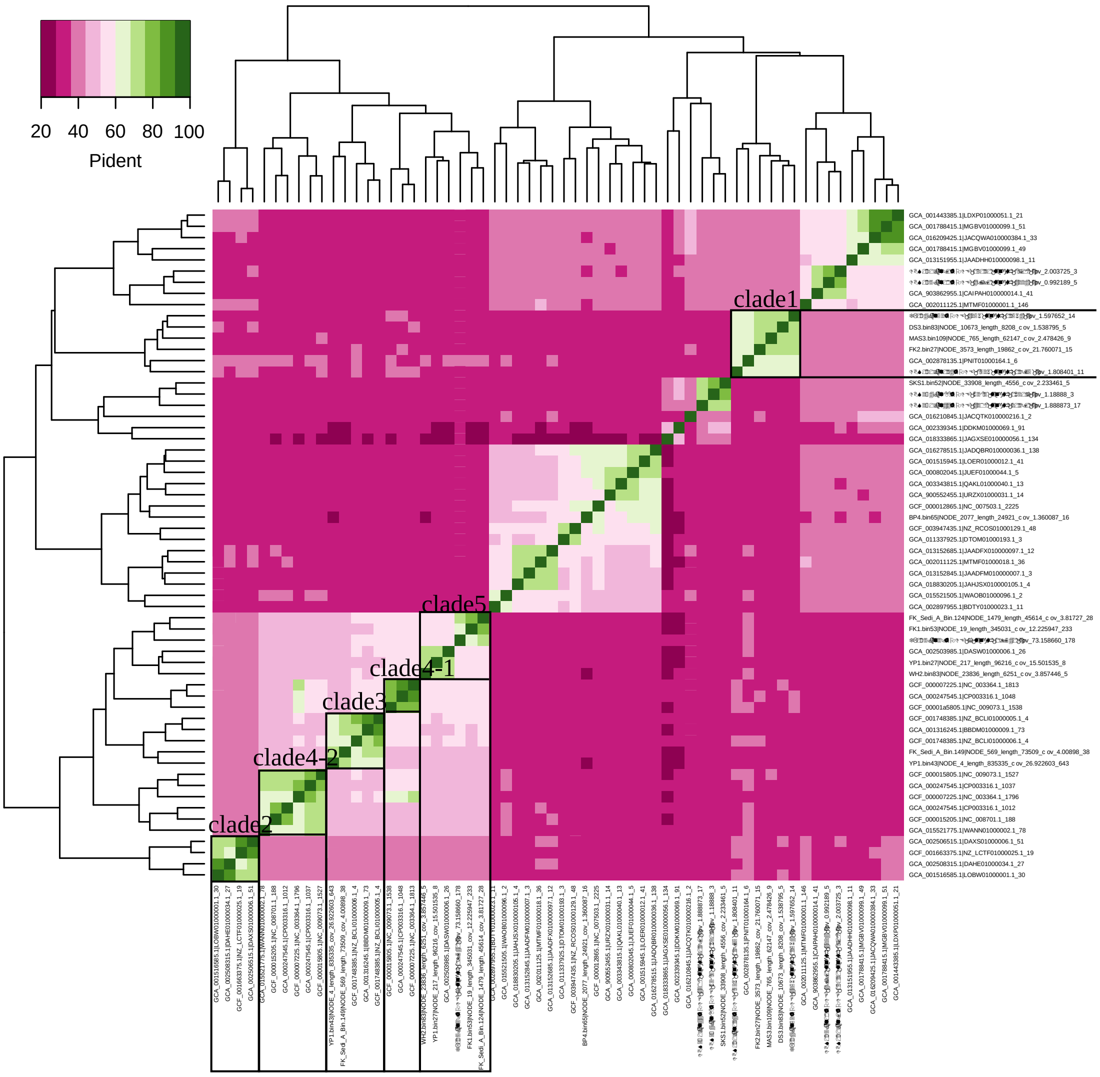

Figure S1 Multiple sequence alignment of RA-DsrAB found in the reconstructed DsrAB tree in Figure 1. Pident represents the percentage of identical matches. Sequences of different clades are marked with black boxes.

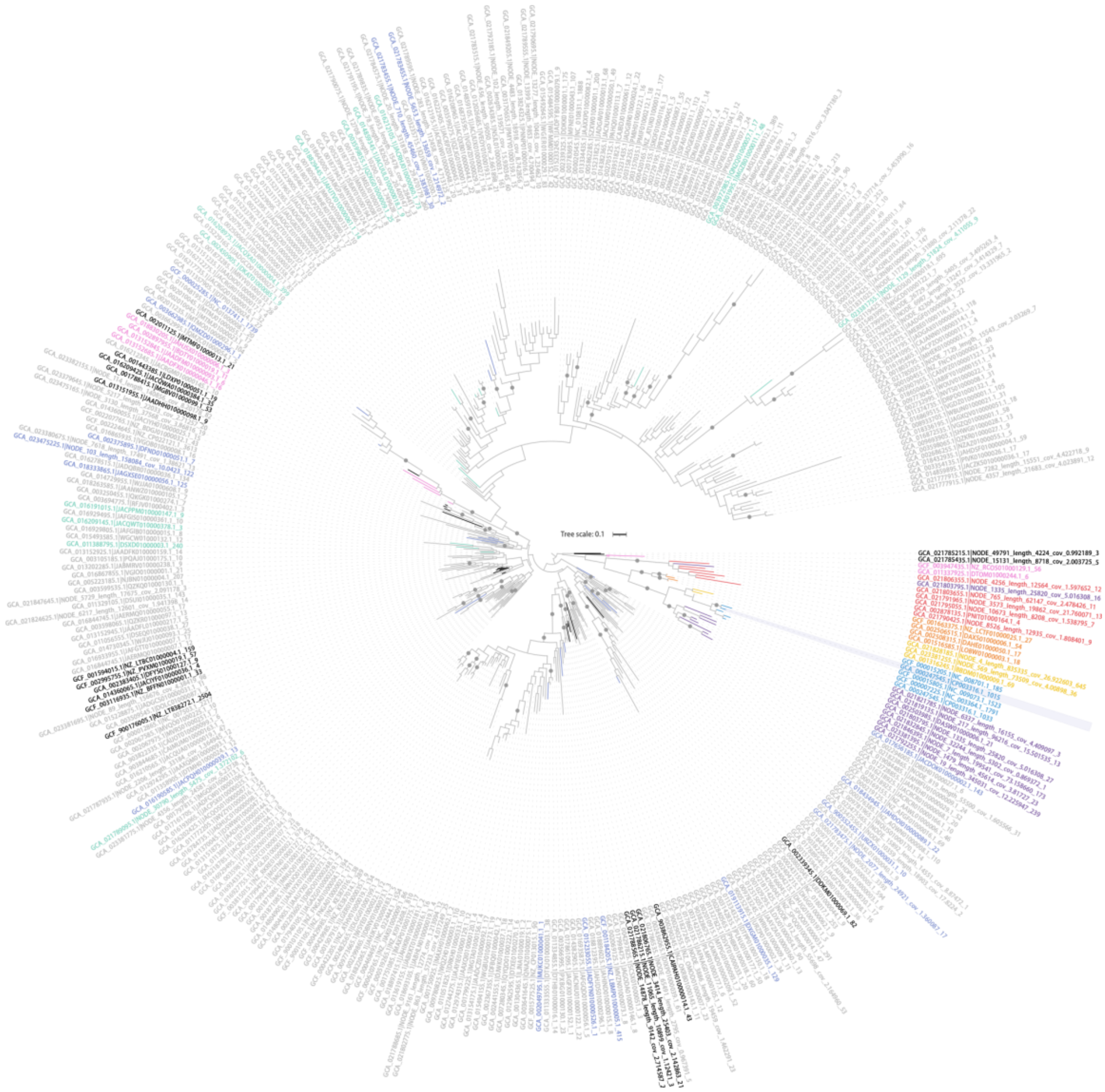

Figure S2 Maximum likelihood phylogenetic tree (Q.yeast + I + R8) of DsrC (372 sequences with 104 amino-acid sites). Branches with ultrafast bootstrap support values ≥ 95% are indicated by black dots. If the protein belongs to the same genome as a DsrAB homolog, it is marked with the same color as the corresponding DsrAB sequence, as shown in Figure 1. The tree is rooted by setting the outgroup as the largest monophyletic clade containing the highest proportion of DsrC homologs sharing the same genomes as DsrAB homologs belonging to basal types, reductive archaeal type, or transitional archaeal type.

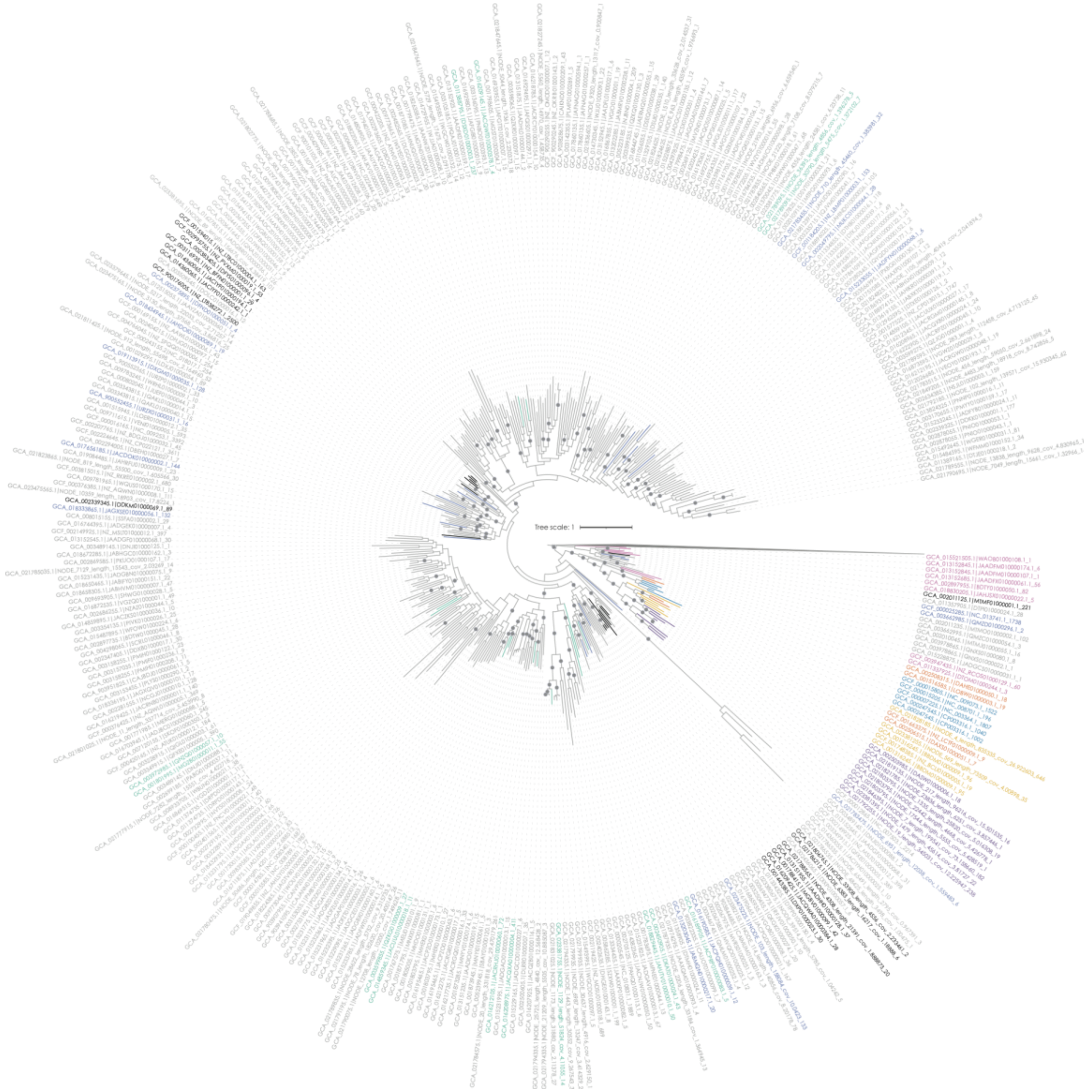

Figure S3 Maximum likelihood phylogenetic tree (Q.pfam + R10) of DsrN (570 sequences with 1121 amino-acid sites). Branches with ultrafast bootstrap support values ≥ 95% are indicated by black dots. If the protein belongs to the same genome as a DsrAB homolog, it is marked with the same color as the corresponding DsrAB sequence, as shown in Figure 1. CbiA, CobB, and CfbB sequences were used as the outgroup (the collapsed gray clade) in rooting.

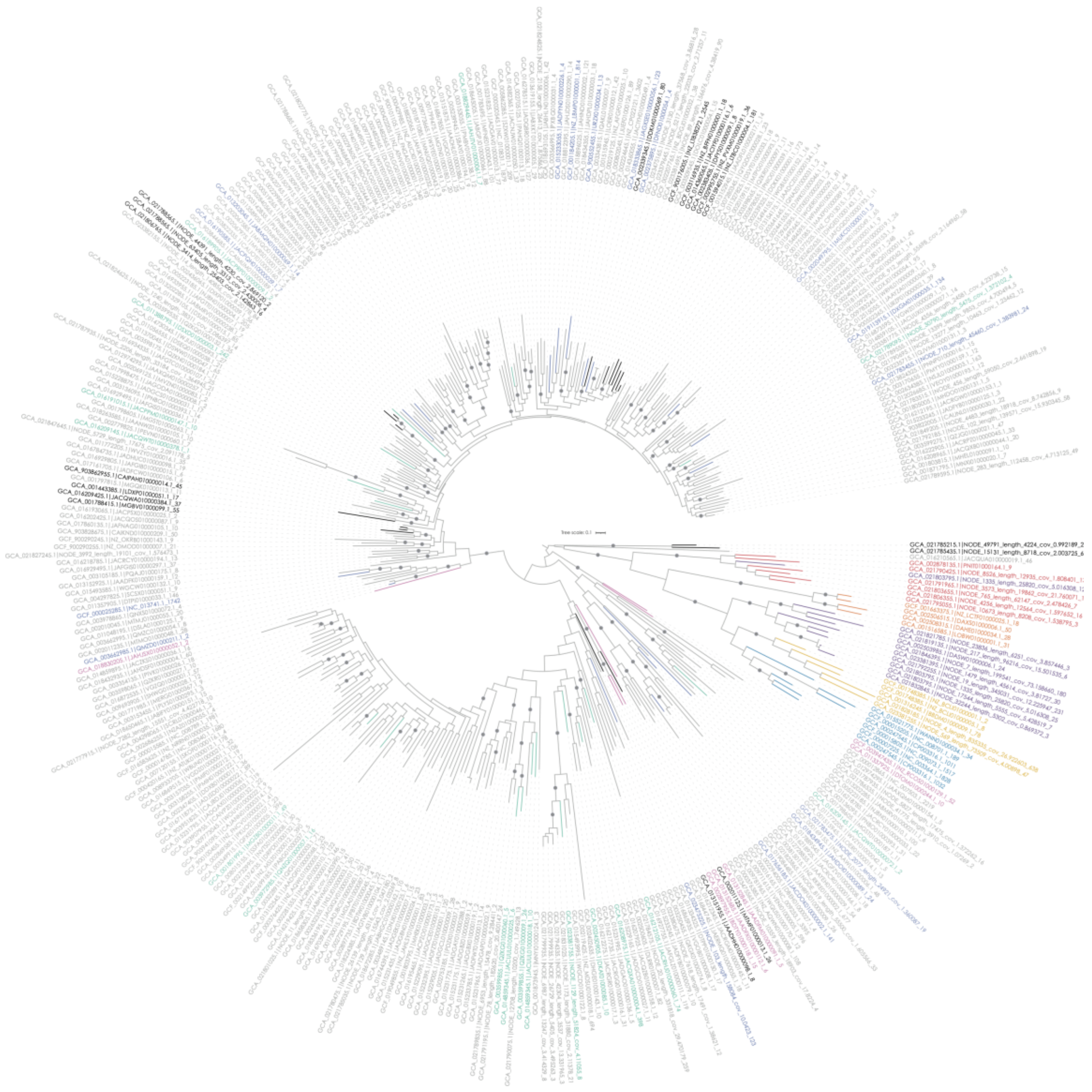

Figure S4 Maximum likelihood phylogenetic tree (LG + F + I + R10) of DsrM (371 sequences with 378 amino-acid sites). Branches with ultrafast bootstrap support values ≥ 95% are indicated by black dots. If the protein belongs to the same genome as a DsrAB homolog, it is marked with the same color as the corresponding DsrAB sequence, as shown in Figure 1. The tree was rooted at the midpoint.

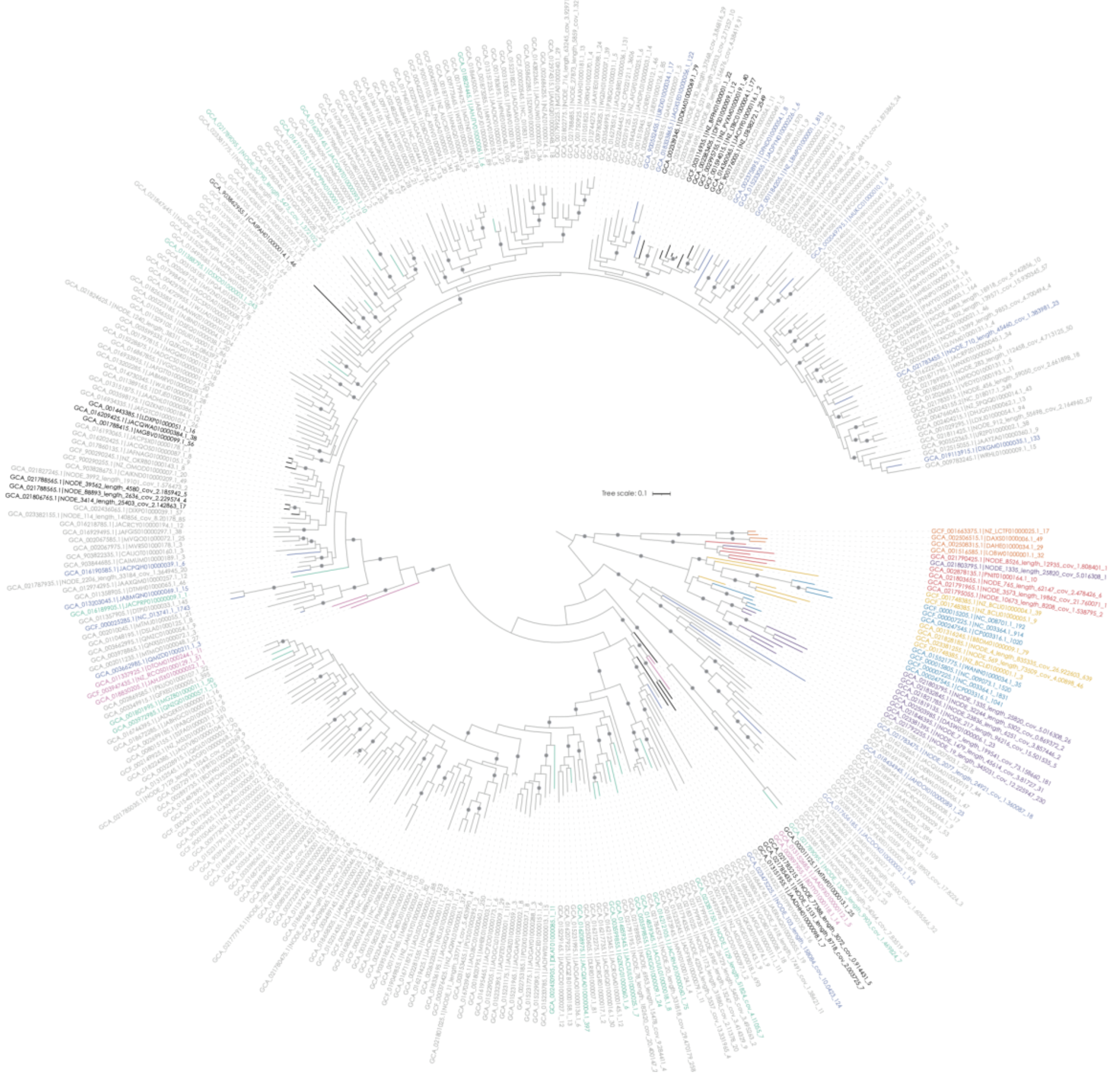

Figure S5 Maximum likelihood phylogenetic tree (LG + I + R9) of DsrK (361 sequences with 479 amino-acid sites). Branches with ultrafast bootstrap support values ≥ 95% are indicated by black dots. If the protein belongs to the same genome as a DsrAB homolog, it is marked with the same color as the corresponding DsrAB sequence, as shown in Figure 1. The tree is rooted at the midpoint.

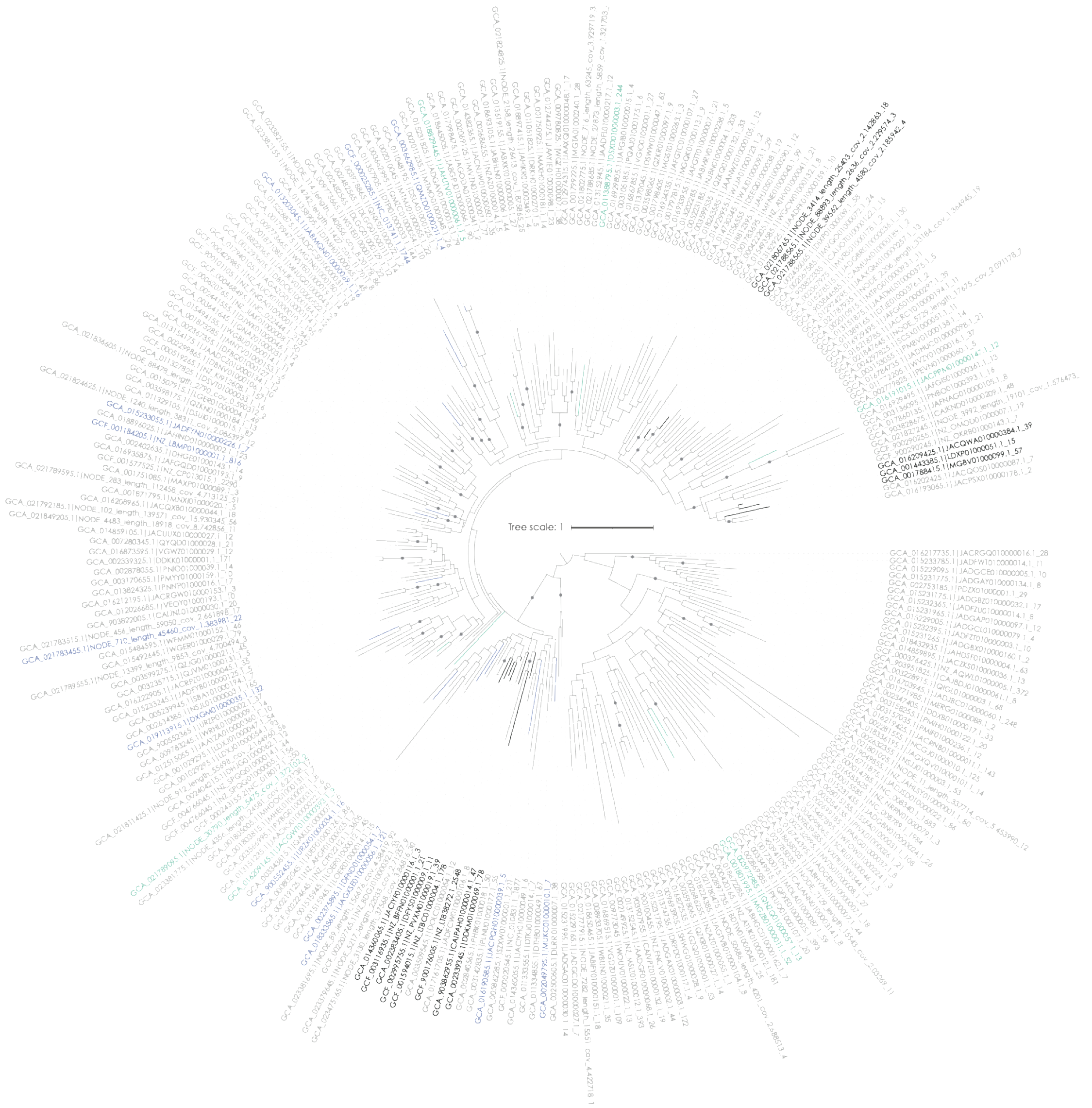

Figure S6 Maximum likelihood phylogenetic tree (WAG + I + R7) of DsrJ (271 sequences with 276 amino-acid sites). Branches with ultrafast bootstrap support values ≥ 95% are indicated by black dots. If the protein belongs to the same genome as a DsrAB homolog, it is marked with the same color as the corresponding DsrAB sequence, as shown in Figure 1. The tree is rooted at the midpoint.

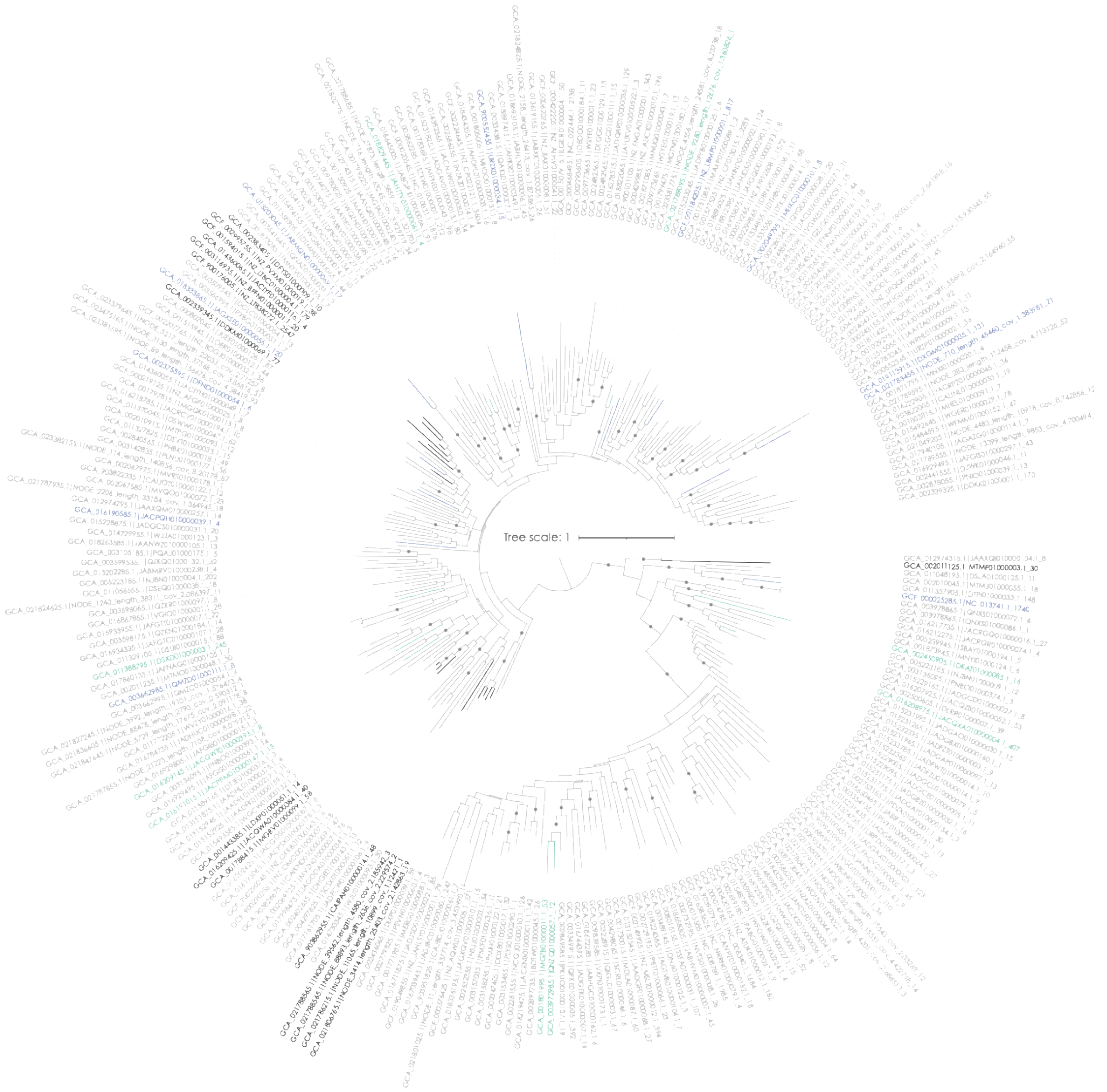

Figure S7 Maximum likelihood phylogenetic tree (Q.pfam + I + R8) of DsrO (278 sequences with 353 amino-acid sites). Branches with ultrafast bootstrap support values ≥ 95% are indicated by black dots. If the protein belongs to the same genome as a DsrAB homolog, it is marked with the same color as the corresponding DsrAB sequence, as shown in Figure 1. The tree is rooted at the midpoint.

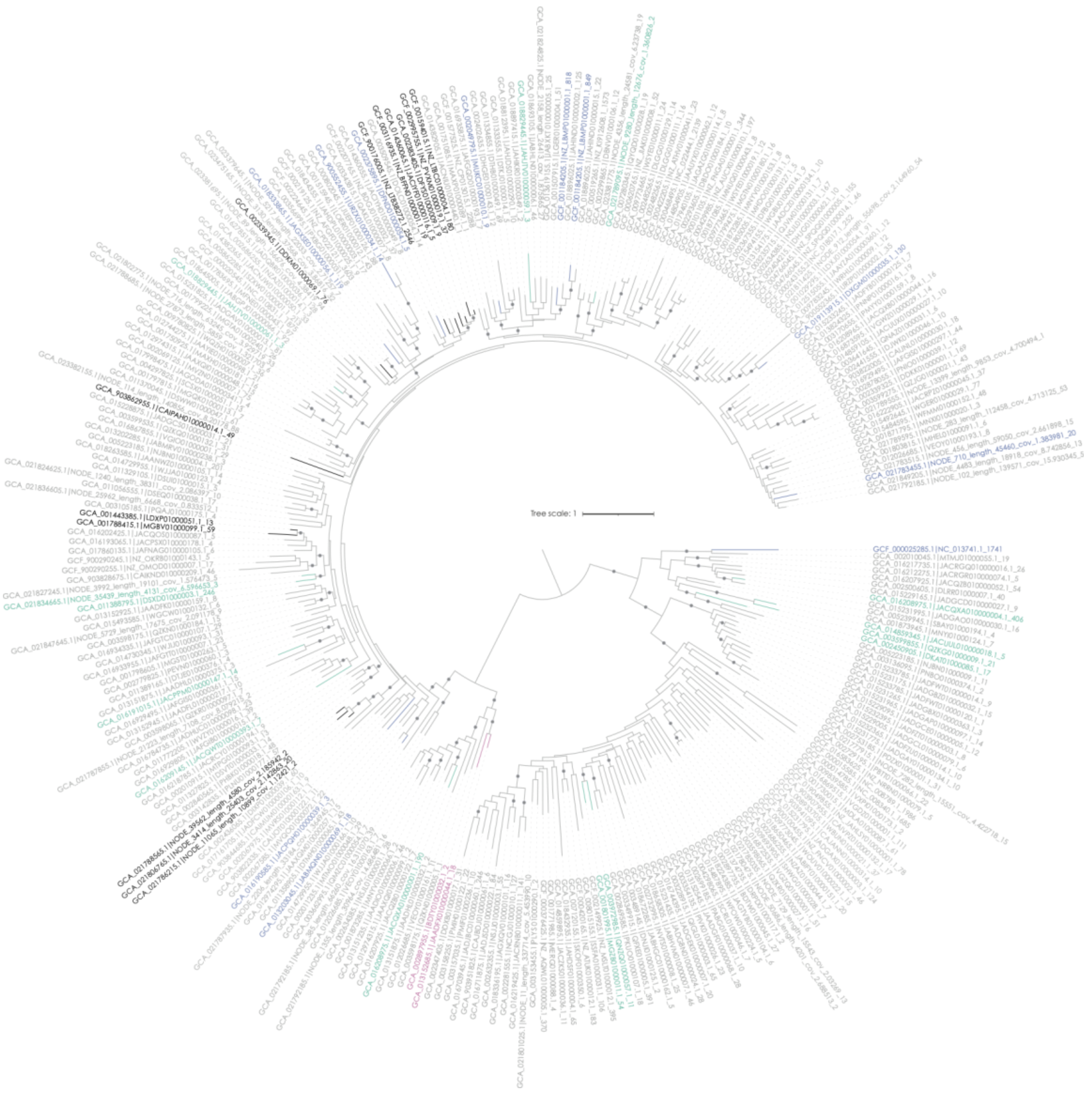

Figure S8 Maximum likelihood phylogenetic tree (LG + F + R9) of DsrP (293 sequences with 550 amino-acid sites). Branches with ultrafast bootstrap support values ≥ 95% are indicated by black dots. If the protein belongs to the same genome as a DsrAB homolog, it is marked with the same color as the corresponding DsrAB sequence, as shown in Figure 1. The tree is rooted at the midpoint.

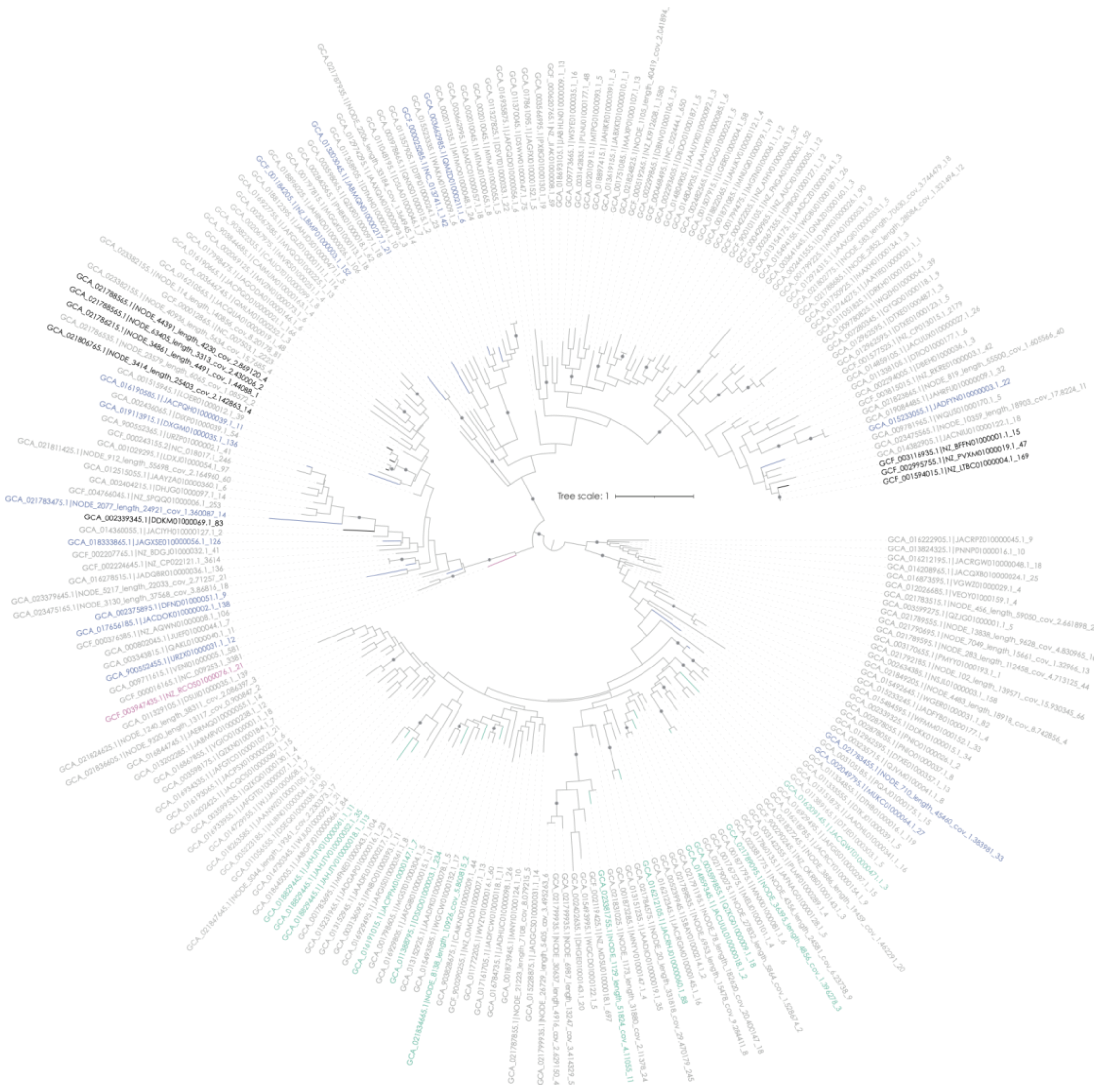

Figure S9 Maximum likelihood phylogenetic tree (Q.yeast+F+R5) of DsrD (227 sequences with 170 amino-acid sites). Branches with ultrafast bootstrap support values ≥ 95% are indicated by black dots. If the protein belongs to the same genome as a DsrAB homolog, it is marked with the same color as the corresponding DsrAB sequence, as shown in Figure 1. The tree is rooted at the midpoint.

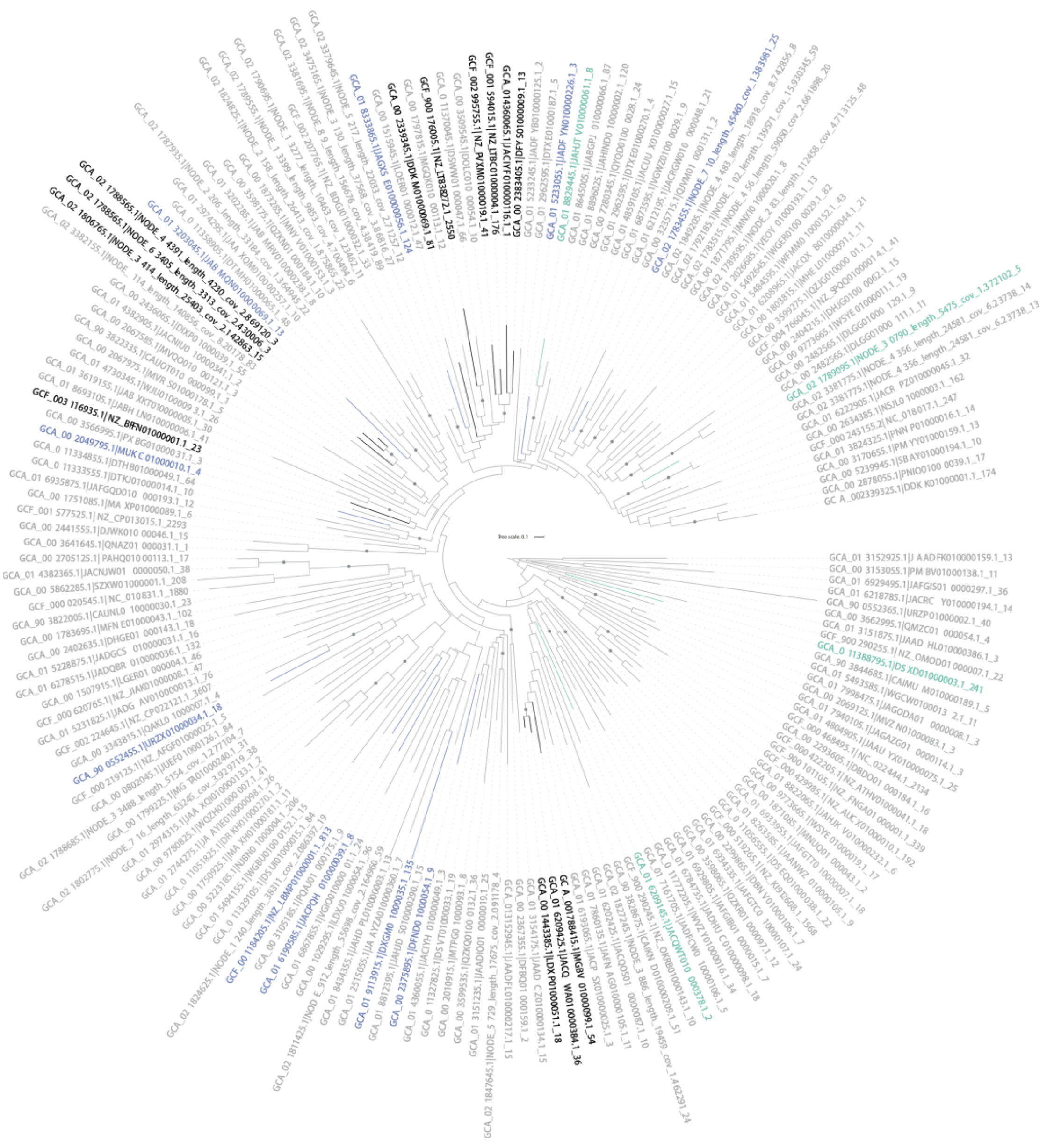

Figure S10 Maximum likelihood phylogenetic tree (Q.pfam + R7) of DsrT (180 sequences with 238 amino-acid sites). Branches with ultrafast bootstrap support values ≥ 95% are indicated by black dots. If the protein belongs to the same genome as a DsrAB homolog, it is marked with the same color as the corresponding DsrAB sequence, as shown in Figure 1. The tree is rooted at the midpoint.

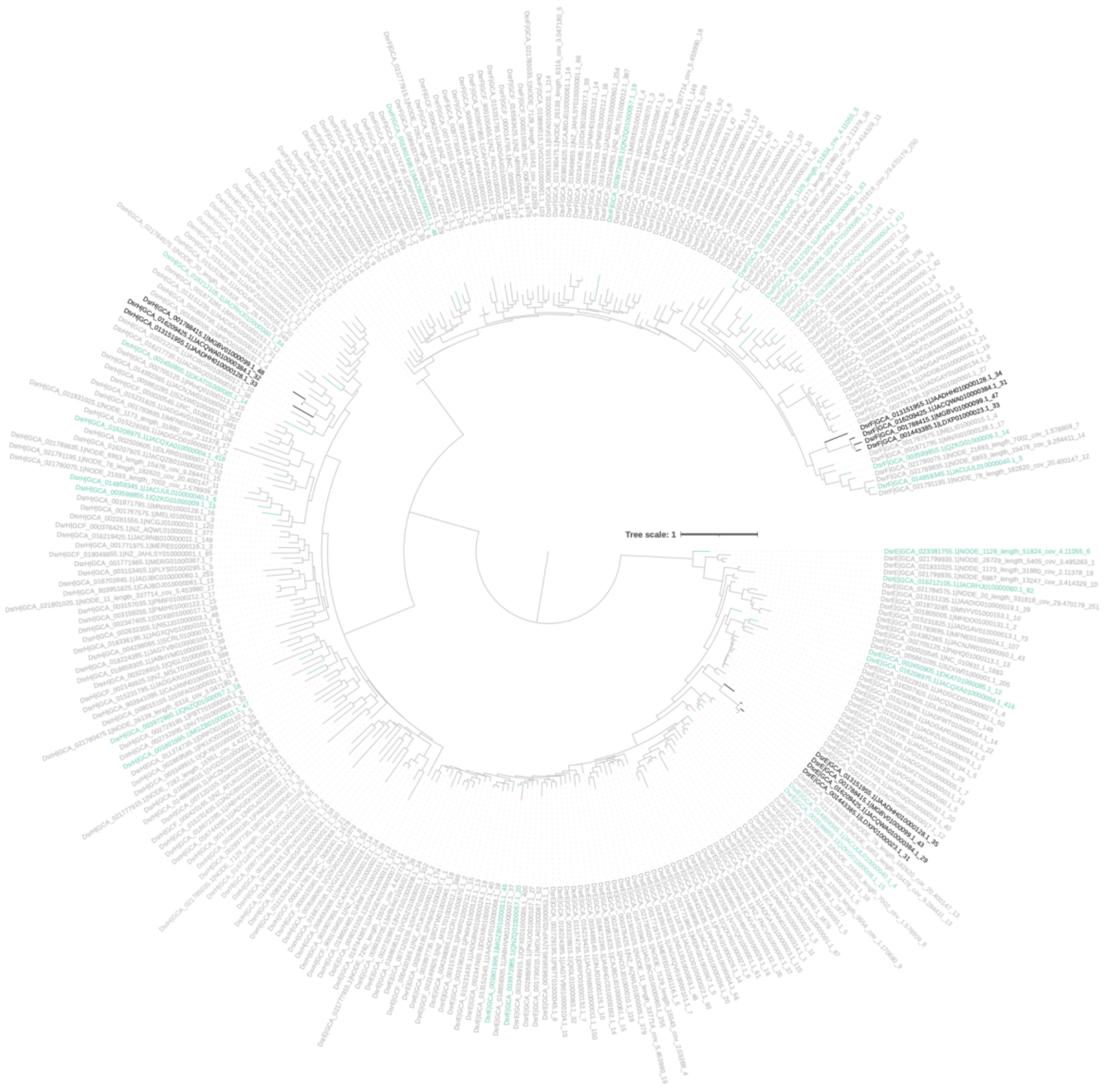

Figure S11 Maximum likelihood phylogenetic tree (Q.pfam + I + R6) of DsrE, DsrF, and DsrH (303 sequences with 98 amino-acid sites). Branches with ultrafast bootstrap support values ≥ 95% are indicated by black dots.If the protein belongs to the same genome as a DsrAB homolog, it is marked with the same color as the corresponding DsrAB sequence, as shown in Figure 1. The tree is rooted using the paralogous rooting method.

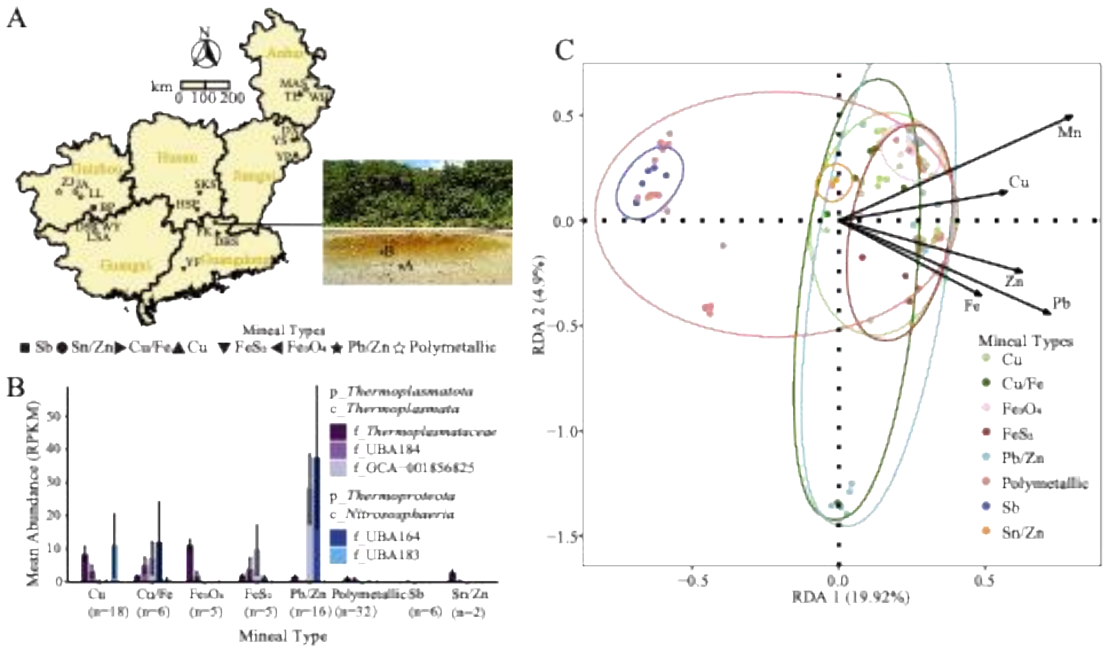

Figure S12 An overview of AMD sediment samples and *dsrA/dsrB*-carrying archaeal MAGs. A Sampling site of acid mines in six provinces in southern China, marked by different shapes indicating mineral types. Two tailings samples were collected at the FK site (A and B), showing the surrounding environment. **B** Abundance of *dsrA/dsrB*-carrying MAGs in 90 AMD sediment samples. **C** Redundancy analysis (RDA) showing the community structure of *dsrA/dsrB*-carrying archaea in response to physicochemical factors. Sample sites are shown as dots colored by their mineral type. The ellipses in the figure reflect the distribution of samples of different mineral types with 95% confidence.

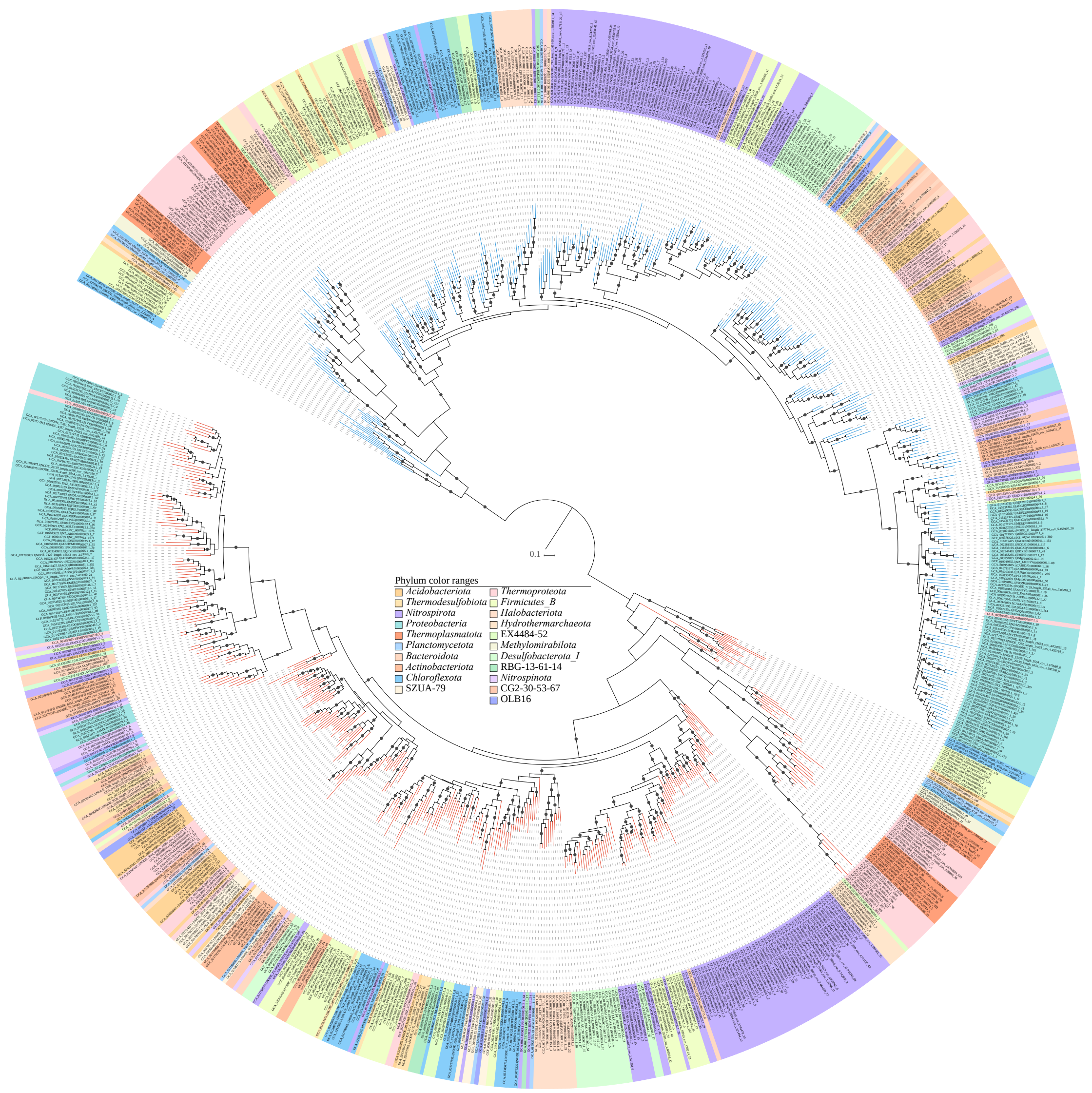

Figure S13 Paralogous rooting of the DsrA and DsrB subunits using 415 concatenated DsrAB sequences. The maximum likelihood phylogenetic tree (LG + I + R10) showing the phylogenetic relationship between DsrA and DsrB (830 sequences, 716 amino-acid sites). The branches with ultrafast bootstrap support values ⩾ 95% are marked with black dots. The red branches show DsrA, and the blue ones show DsrB.

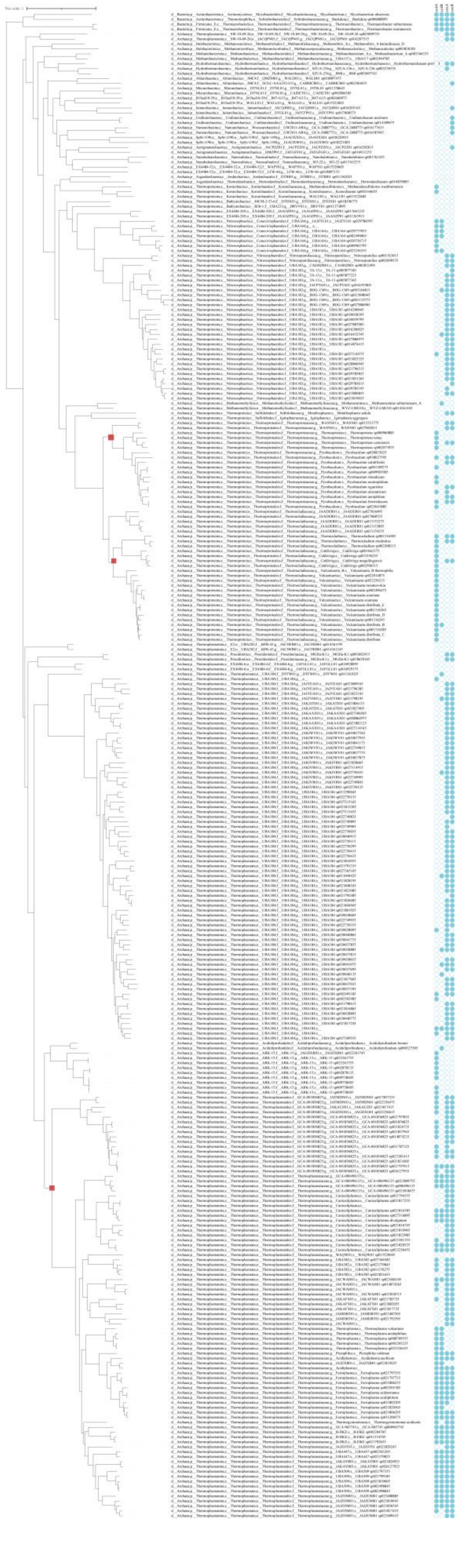

Figure S14 Distribution of cytochrome oxidase genes and cytochrome bd quinol oxidase genes in the species tree. The maximum likelihood phylogenetic tree (LG+PMSF+F+G, 12166 alignment positions) is the same as Figure 2 and Figure 3. The presence of cytochrome oxidase genes and cytochrome bd quinol oxidase genes was mapped onto the tree with blue dots. The internal nodes used for molecular dating were marked with red squares.

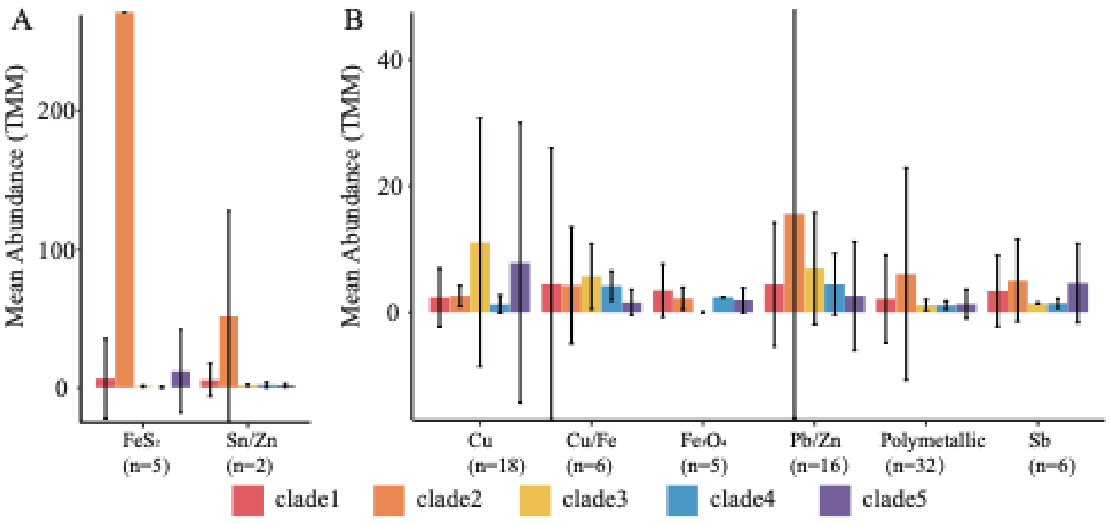

Figure S15 Abundances of RA-DsrAB clades in AMD sediment samples of different mineral types. Means and standard deviations of abundances are shown. **A** Mineral type FeS_2 and Sn/Zn. **B** Mineral type Cu, Cu/Fe, Fe_3O_4, Pb/Zn, Polymetallic, and Sb.

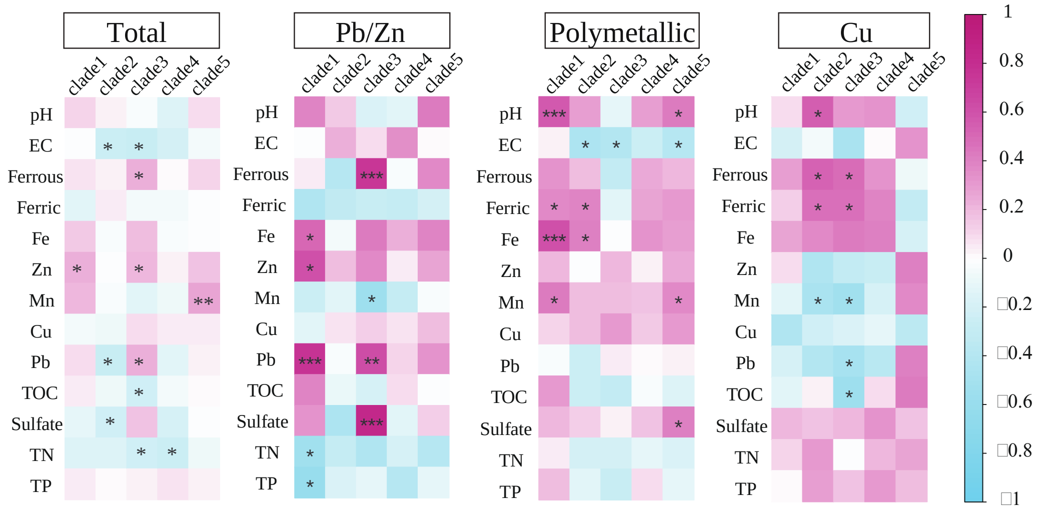

Figure S16 The correlations between gene abundance of five RA-DsrAB clades and abiotic variables in all Pb/Zn, Polymetallic, and Cu mineral types of AMD sediment samples. The color gradient in the heatmap denotes Spearman's correlation coefficients, and the asterisk indicates a two-tailed test of Spearman's statistical significance. *$P < 0.05$, **$P < 0.01$ and ***$P < 0.001$. EC, electronic conductivity; TOC, total organic carbon; TN, total nitrogen; TP, total phosphorus; TS, total sulfur.
